# Multi-cancer analysis reveals universal association of oncogenic LBH expression with DNA hypomethylation and WNT-Integrin signaling pathways

**DOI:** 10.1038/s41417-023-00633-y

**Published:** 2023-06-02

**Authors:** In-Chi Young, Thomas Brabletz, Linsey E. Lindley, Maria Abreu, Nagaraj Nagathihalli, Alexander Zaika, Karoline J. Briegel

**Affiliations:** 1 Department of Surgery, Division of Surgical Oncology, University of Miami Miller School of Medicine, Miami, FL USA; 2 Braman Family Breast Cancer Institute, Sylvester Comprehensive Cancer Center, University of Miami Miller School of Medicine, Miami, FL USA; 3 Department of Experimental Medicine 1, Nikolaus-Fiebiger-Center for Molecular Medicine, Friedrich-Alexander-University Erlangen-Nürnberg, Erlangen, Germany; 4 Graduate Program in Biochemistry and Molecular Biology, University of Miami Miller School of Medicine, Miami, FL USA; 5 Department of Medicine, Division of Gastroenterology, University of Miami Miller School of Medicine, Miami, FL USA

**Keywords:** Tumour biomarkers, Biomarkers

## Abstract

Limb-Bud and Heart (LBH) is a developmental transcription co-factor deregulated in cancer, with reported oncogenic and tumor suppressive effects. However, LBH expression in most cancer types remains unknown, impeding understanding of its mechanistic function Here, we performed systematic bioinformatic and TMA analysis for LBH in >20 different cancer types. LBH was overexpressed in most cancers compared to normal tissues (>1.5-fold; p < 0.05), including colon-rectal, pancreatic, esophageal, liver, stomach, bladder, kidney, prostate, testicular, brain, head & neck cancers, and sarcoma, correlating with poor prognosis. The cancer types showing LBH downregulation were lung, melanoma, ovarian, cervical, and uterine cancer, while both LBH over- and under-expression were observed in hematopoietic malignancies. In cancers with LBH overexpression, the *LBH* locus was frequently hypomethylated, identifying DNA hypomethylation as a potential mechanism for LBH dysregulation. Pathway analysis identified a universal, prognostically significant correlation between *LBH* overexpression and the WNT-Integrin signaling pathways. Validation of the clinical association of LBH with WNT activation in gastrointestinal cancer cell lines, and in colorectal patient samples by IHC uncovered that LBH is specifically expressed in tumor cells with nuclear beta-catenin at the invasive front. Collectively, these data reveal a high degree of LBH dysregulation in cancer and establish LBH as pan-cancer biomarker for detecting WNT hyperactivation in clinical specimens.

## Introduction

Cancer is a major public health problem and leading cause of human death worldwide [1]. Despite the advancement of novel combined treatment strategies and expanded understanding of the underlying mechanisms, the mortality rate of cancer patients remains high [1]. Identification of pan-cancer biomarkers for predicting patient survival or targetable signaling pathways, therefore, represents an unmet medical need.

Limb-Bud and Heart (LBH) is a highly conserved, tissue-specific transcription cofactor in vertebrates [2–4] involved in development and misregulated in cancer. In embryonic development, changes in *LBH* gene dosage affect heart morphogenesis, bone formation, progenitor cell proliferation/differentiation, angiogenesis [2, 3, 5], as well as neural crest cell migration and gastrulation in fish and frog [6, 7]. LBH is also expressed in adult tissues [3, 8], and *Lbh* gene knockout studies in mice have indicated it is critical for stem cell self-renewal in the postnatal mammary gland [9, 10], inner ear hair cell maintenance [11], and for preventing inflammation [12].

The first evidence that LBH is deregulated in cancer was provided by Rieger et al. by showing LBH is aberrantly overexpressed in worst prognosis, treatment-resistant basal-like breast cancers [13]. Notably, *LBH* is a direct target gene of the oncogenic WNT/ß-catenin signaling pathway, and genetic studies, showing *Lbh* knockout attenuates WNT-induced mammary tumorigenesis [14], imply an important role for LBH in WNT-driven cancers.

Prognostically significant LBH overexpression has also been reported in hepatocellular [15], gastric cancers [16, 17], and glioma [18, 19], where it promotes cell proliferation, invasion, angiogenesis, and tumor growth, in part through FAK/PI3K/AKT- and/or VEGF/ERK-dependent mechanisms [16, 18]. Cell-based transcriptional reporter assays in non-cancer cells suggest LBH increases the activity of transcription factor oncogene, AP1 [8], while it appears to repress promoter activity of tumor suppressor genes, p53 and p21 [20], supportive of an oncogenic role.

In contrast, LBH has been shown to be downregulated in nasopharyngeal and lung cancer, and to exert tumor-suppressive, non-invasive effects, in part by inducing G1/S cell cycle arrest downstream of TGFß and attenuating NF-κB transcriptional activity [21–23]. However, the expression and function of LBH in most cancers remain unknown.

Here, we performed a systematic meta-analysis of *LBH* expression in a wide range of cancers, querying association with patient survival, DNA methylation status, and targetable signaling pathways, with immunohistochemical validation in patient samples and studies in multi-cancer cell lines, to explore the potential of LBH as a pan-cancer diagnostic marker and investigate potential mechanisms underlying the LBH dysregulation in cancer.

## Materials and methods

### mRNA expression analysis

Data regarding *LBH* mRNA expression in different cancer types relative to normal tissues were retrieved from the Oncomine 4.5 database (https://www.oncomine.org/resource/login.html) [24]. The threshold parameters were set at *p* < 0.05; fold-change > 1.5. To confirm expression profiles and determine association with tumor stages, we extracted *LBH* mRNA expression data from the Gene Expression Profiling Interactive Analysis, GEPIA2 (http://gepia2.cancer-pku.cn), an interactive online platform for integrating RNA sequencing data from The Cancer Genome Atlas (TCGA, https://www.cancer.gov/tcga) [25] and the Genotype-Tissue Expression (GTEx) project of normal tissues [26].

### Survival analysis

The correlations between *LBH* expression and patient survival in different cancer types were determined using PROGgene V2 (http://genomics.jefferson.edu/proggene) [27], R2 platform (http://r2platform.com) [28], Kaplan–Meier plotter (http://kmplot.com/analysis/) [29], GEPIA2, and individual microarray datasets from TCGA and the Gene Expression Omnibus (GEO) database (https://www.ncbi.nlm.nih.gov/geo/). Integrated survival curves for *LBH* and WNT/Integrin pathway genes were constructed with PROGgene V2. The optimum cut-off values divided *LBH* expression into high and low-expression groups based on p-values from log-rank test for each dataset. Significant Kaplan–Meier survival plots with log rank test (hazard ratios at 95% confidence interval) and *p*-values < 0.05 are illustrated.

### Gene alteration/mutation analysis

*LBH* gene alteration patterns and frequencies across multiple cancer studies were determined using cBioPortal (http://www.cbioportal.org) [30, 31].

### DNA methylation analysis

The DNA methylation status of the *LBH* gene locus, and their prognostic values in cancers were analyzed by MethSurv (https://biit.cs.ut.ee/methsurv/) [32], using TCGA data. For survival analysis of *LBH* methylation at specific CpG sites, the optimal high/low methylation cutoff points were defined as the ones with the most significant split. Heatmaps for *LBH* DNA methylation levels were generated using the gene visualization feature of MethSurv. For correlation of *LBH* mRNA expression and DNA methylation level, cBioportal was employed to calculate the Pearson coefficient and draw correlation plots.

### Gene correlation analysis

The R2 platform (http://r2platform.com) [28] was used to identify *LBH*-correlated genes in colorectal, stomach, liver, pancreatic, esophageal, kidney, bladder, prostate, brain, lung cancers, and melanoma using TCGA datasets. *LBH* co-expressed genes were identified with Bonferroni correction (*p*-value < 0.01). To generate common gene lists positively correlated with *LBH*, we utilized the Venn diagram generator, Venny 2.1 [33]. Gene sets were then evaluated for enriched pathways using the web-based Protein Analysis Through Evolutionary Relationships (PANTHER) tool (http://pantherdb.org/) [34], or the Kyoto Encyclopedia of Genes and Genomes (KEGG 101.0) [35]. The co-expression profiles of *LBH* with WNT and Integrin pathway genes were extracted from Oncomine and depicted as heatmaps. The correlation plots of *LBH* with individual WNT pathway genes in colon, stomach, and pancreatic cancer were generated using cBioportal.

### Immunohistochemistry analysis

Tissue microarrays (BCN721b, MC482), containing 21 different human cancer types and corresponding normal tissues from a total of 112 patients were purchased from TissueArray.Com LLC, USA. Normal skin and melanoma paraffin tissue sections were obtained from the Cancer Modeling Shared Resources of the Sylvester Comprehensive Cancer Center, University of Miami. Colorectal cancer tissue samples used in the present study (all cases were G2 or G3 adenocarcinomas) were retrieved from the archives of the Department of Pathology, University Erlangen. These samples were from patients operated in 2003 or earlier. Patient identity was anonymized, an informed consent was not required at that time. The usage for immunohistochemical analyses of the samples was approved by the local ethics committee (approval 374-14). Paraffin sections (3 µm) were deparaffinized in Xylene, rehydrated in Ethanol series and pretreated by boiling in 10 mM citrate buffer pH 6.0 in a pressure cooker. Sections were incubated 15 min at RT with 3% peroxidase, followed by 3 × 5 min washes in TBST, and overnight incubation at 4 °C with IHC validated anti-LBH antibody [9, 10] at a dilution of 1:150–1:1000 in DAKO dilution buffer (DAKO; S3022) or with anti-β−catenin antibody (Sigma; 1:750). Slides were then washed 3× in TBST, incubated 60 min at RT with secondary antibody (DAKO: K4003-HRP), followed by 3× washes in TBST, 20-30 min chromogen AEC, dehydration, and counterstaining with hematoxylin.

### Cell lines

All cell lines were from the American Type Culture Collections (ATCC) and cultured according to the manufacturer’s recommendation.

### Methylation-specific qPCR

Cells were lysed with proteinase K (50 µg/ml, Thermo) at 55 °C overnight and genomic DNA (gDNA) extracted with QIAquick PCR purification kit (QIAGEN), followed by Bisulfite conversion using the gDNA EpiJET Bisulfite conversion kit (Thermo). The primer sets used for methylated or unmethylated gDNA amplifications at CpG sites upstream of the *LBH* promoter were designed with MethPrimer (https://www.urogene.org/cgi-bin/methprimer/methprimer.cgi). qPCR analysis was performed in triplicates with two biological samples. Primer sequences are listed in Supplementary Table S7.

### qPCR and Western blot analysis

qPCR analysis for *LBH* was performed as in [13]. Western Blot analysis used 30–50 µg of total cell lysates and primary antibodies to LBH (1:1000; in house), β-catenin (1:2000; BD), TCF4 (1:1000; 6H5-3, Upstate), and β-actin (1:10,000; AC-15; Sigma), followed by incubation with secondary HRP-conjugated IgGs (1:10,000; Invitrogen).

### Luciferase reporter assays

Cells were seeded at 2 × 10^5^ cells/well onto 12-well plates and transfected the next day with 400 ng of either TOPFlash or FOPFlash luciferase reporter plasmids using Lipofectamine 3000 reagent (Invitrogen). Cells were harvested 48 h later and lysates were analyzed for luciferase activity using Promega Veritas luminometer. Fold activation represents the ratio between TOPFlash and FOPFlash activities of three independent experiments with duplicate samples each.

### Small interfering RNA (siRNA)-mediated gene knockdown

Cells in duplicates were transiently transfected with 2 nM of synthetic siRNA specific for *CTNNB1* or a scrambled control sequence (Dharmacon SmartPool) using Dharmafect #1 transfection reagent (Dharmacon) according to the manufacturer’s protocol.

### Statistical analysis

The significance of Oncomine mRNA expression data was determined by unpaired *t*-test comparing the two groups (normal vs. cancer). One-way ANOVA test was used for the *LBH* differential expression in various cancer types in GEPIA2, and for tumor stage analysis. All survival curves constructed with GEPIA2, R2 platform, PROGgene V2, Kaplan–Meier plotter, and cBioportal were analyzed by log-rank test. The *p*-values for *LBH* DNA methylation levels among different cancer types were calculated using the Wilcoxon rank sum test. The *p*-values for the pathway enrichment analysis were calculated with Fisher exact test. *P*-values < 0.05 were considered significant.

## Results

### LBH is overexpressed in majority of cancer types relative to normal tissues

**Fig. 1:**
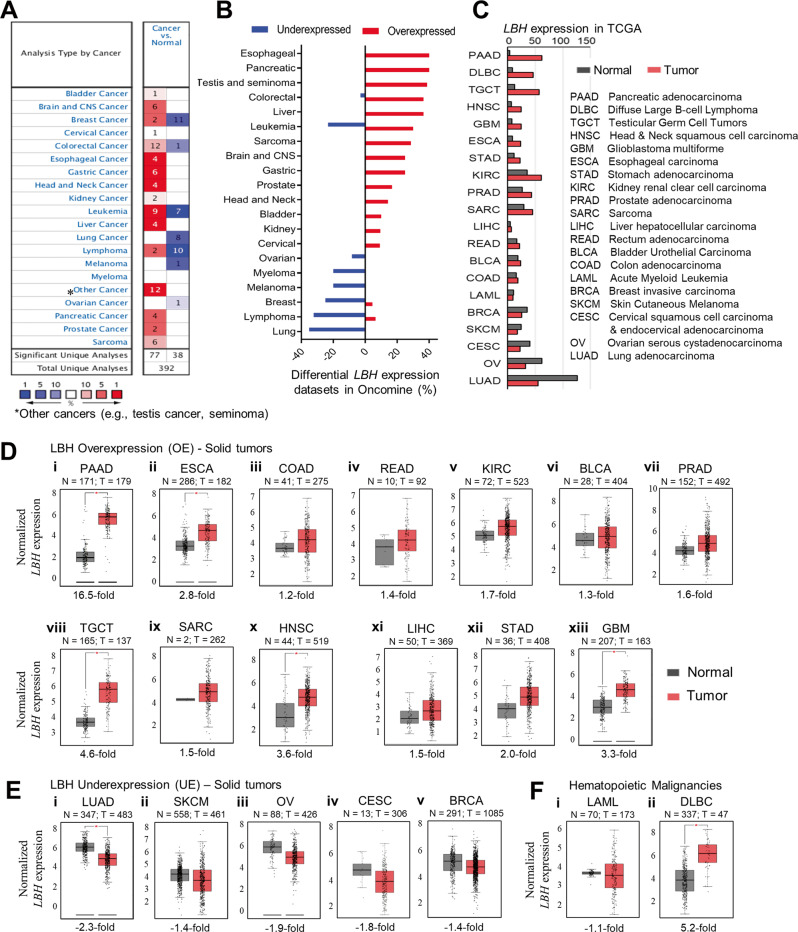
Meta-analysis of *LBH* expression in different human cancers. **A** The differential expression of *LBH* mRNA among various cancer types compared to the corresponding normal tissue was generated using Oncomine. The number of datasets with statistically significant (*p* < 0.05; >1.5-fold change) *LBH* overexpression (red) or underexpression (blue) are shown. The color scale at the bottom represents the percentages of *LBH* gene expression ranking in a specific cancer type compared to normal tissue. Dark red, red, and pink indicate that *LBH* was among the top 1%, top 5%, or top 10%, respectively, of upregulated genes in a dataset. Dark blue, blue, and light blue indicate that *LBH* was among the top 1%, top 5%, or top 10% of downregulated genes. White indicates no significant changes in *LBH* gene expression levels. **B** The percentages of overexpressed and underexpressed analyses for *LBH* in Oncomine were calculated and plotted in the order of differential *LBH* expression percentage. **C** Column plot showing *LBH* mRNA levels in tumor versus normal tissue in different cancer types in The Consortium Genome Atlas (TCGA) database in descending order of *LBH* expression (fold change). The abbreviation for each cancer type is explained in the table on the right. Pancreatic cancer (PAAD) showed the highest, and lung cancer (LUAD) the lowest *LBH* expression compared to normal tissues. **D**–**F** Box plots generated from TCGA data (in **C**) showing the fold changes of *LBH* mRNA in tumor (red) versus normal tissues (gray). **D** Solid tumors with *LBH* overexpression; **E** solid tumors with *LBH* underexpression; and **F** blood cancers with deregulated *LBH* expression. The threshold was set at *p*-value = 0.05. The number of normal (N) and tumor (T) tissues is indicated for each cancer type. *LBH* expression data were extracted from the TCGA and GTEx databases using GEPIA2. See also Fig. S1.

To assess the degree of LBH dysregulation in cancer, we examined its mRNA expression in all available cancer gene expression data using the visualization tools from the Oncomine gene expression database (Fig. 1A, B). Differential *LBH* expression in cancer compared to normal tissue (fold change >1.5; *p*-value < 0.05) was detected in over 20 different cancer types (Fig. 1A, B). Since The Cancer Genome Atlas (TCGA) is the landmark of cancer genomics with an extensive collection of open-source RNA sequencing data, we compared the *LBH* expression profiles from Oncomine with data in TCGA via GEPIA2 (Fig. 1C). This analysis confirmed *LBH* overexpression in fourteen, and *LBH* underexpression in six common cancer types, with > 90% consensus between the two databases (Fig. 1A–C).

*LBH* was significantly (*p* < 0.05) and consistently overexpressed relative to normal tissue in: pancreatic adenocarcinoma (PAAD; +2.9 to 16.5-fold), esophageal carcinoma (ESCA; +2.8 to 2.9-fold), colon adenoma (COAD; +1.2 to 1.6-fold), rectum adenoma (READ; +1.4 to 2.3-fold), kidney renal clear cell carcinoma (KIRC; +1.5 to 1.7-fold), bladder urothelial carcinoma (BLCA; +1.3 to 1.6-fold), prostate adenocarcinoma (PRAD; +1.6 to 1.9-fold), testicular germ cell tumors (TGCT; +4.6 to 5.7-fold), sarcoma (SARC; +1.5 to 2.4-fold), and head & neck squamous cell carcinoma (HNSC; +2.0 to 3.6-fold) (TCGA data: Fig. 1C, and Fig. 1D.i–x; Oncomine Data: Fig. S1A.i–x). Moreover, *LBH* was upregulated in liver hepatocellular carcinoma (LIHC; +1.5 to 2.5-fold), stomach adenocarcinoma (STAD; +1.8 to 2.0-fold), and in the aggressive brain cancer, glioblastoma (GBM; +1.7 to 3.3-fold) (Fig. 1D.xi–xiii; Fig. S1A.xi–xiii), congruous with previous reports [15–19]. In addition, TCGA analysis revealed *LBH* overexpression in rare bile duct (cholangiocarcinoma/CHOL; +11.8-fold), neuroendocrine (pheochromocytomas and paragangliomas/PCPG; +4.3-fold), and thymus (thymoma/ THYM; +4.6-fold) cancers (Fig. S1B.i–iii).

In contrast, *LBH* was most prevalently downregulated in lung adenocarcinoma (LUAD, −2.3 to −2.5; Fig. 1E.i, and Fig. S1A.xiv), and lung squamous cell carcinoma (LUSC, −2.4-fold; Fig. S1B.iv), confirming published data [22]. Significant *LBH* underexpression was further detected in skin cutaneous melanoma (SKCM, −1.4 to −2.1-fold), ovarian serous cystadenocarcinoma (OV, −1.5 to −1.9-fold), and, in the larger TCGA dataset, also in cervical squamous cell carcinoma (CESC, −1.8-fold), and in uterine-endometrial (UCEC/Uterine Corpus Endometrial Carcinoma; −2.3-fold) cancers (Fig. 1E.ii–iv; Fig. S1A.xv–xvi, and Fig. S1B.v). Lastly, *LBH* was underexpressed in invasive breast carcinoma (BRCA, −1.4 to 2.5-fold), although two datasets in Oncomine also reported *LBH* overexpression (Fig. 1A–C, and Fig. 1E.v; Fig. S1A.xvii), consistent with our previous results [13]. Information on additional histological subtypes within *LBH* over- and under-expressing solid tumors from Oncomine is provided in Supplementary Tables S1 and S2, respectively.

Among the top twenty cancer types with differential *LBH* expression were also blood cancers (Fig. 1A–C). Hence, we next examined *LBH* gene expression in hematopoietic malignancies, which has not yet been explored. We were particularly intrigued by almost equal *LBH* over- and under-expression in leukemia, as suggested by our Oncomine analysis (Fig. 1A, B). Interrogation of individual datasets in Oncomine and TCGA revealed that *LBH* was highly overexpressed in B-cell acute lymphoblastic leukemia (B-ALL, +37.7-fold; Fig. S1A.xviii), the most common and aggressive leukemia subtype, and in chronic lymphocytic leukemia (CLL, +2.8-fold) (Table S3). In contrast, *LBH* was underexpressed in more indolent, late-onset acute myeloid leukemia (LAML/AML, −1.1-fold in TCGA; −2.5-fold in Oncomine) (Fig. 1C; Fig. 1F.i; and Fig. S1A.xix), and in slow-growing, chronic myeloid leukemia (CML, −2.47) and chronic adult T-cell leukemia/lymphoma (ATLL, −2.27) (Table S3). In lymphoma, *LBH* was predominantly underexpressed (Fig. 1A, B, and Table S3). However, it was overexpressed in most common Diffuse Large B-Cell Lymphoma (DLBC; +5.2-fold) (Fig. 1C, and Fig. 1F.ii), and in Follicular Lymphoma (+3.4-fold; Table S3). Hence, in blood cancers *LBH* is both over- and under-expressed, depending on the subtype.

**Fig. 2:**
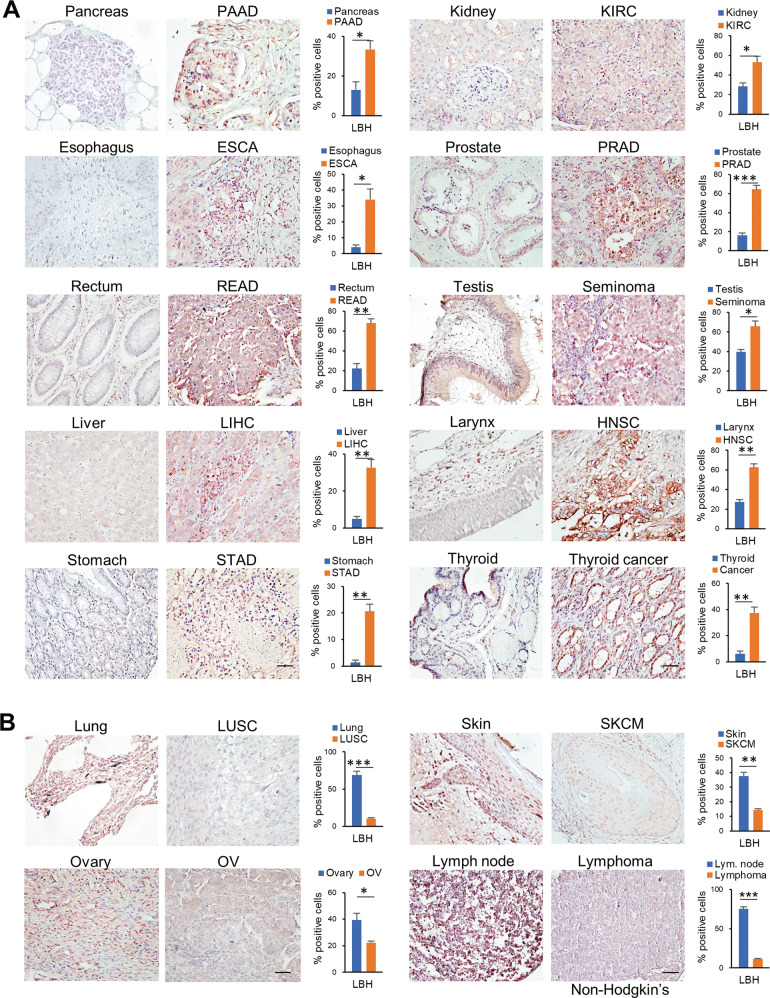
LBH protein expression in human cancer and normal tissues. **A**, **B** Tissue microarray analysis with representative IHC images showing LBH protein expression (brown) in different human cancers compared to normal tissues, as indicated. Hematoxylin (blue) served as nuclear counterstain. **A** Gastrointestinal (left panel), urogenital, head and neck, and thyroid cancers (right panel) with LBH overexpression. **B** Cancer types with decreased LBH protein expression compared to normal tissue. Scale bars: 50 µm. The graphs on the right of each normal-tumor set represent the quantification of LBH-positively stained cells in the nucleus and perinucleus region, expressed as the percentage of total cells. All data are mean ± SEM (*n* = 3 samples for each group). *P*-values; Student *t*-test.

To determine whether the observed changes in *LBH* mRNA expression in the different tumor types translated into similar changes in LBH protein, we performed multiorgan tissue microarray analysis (TMA), using an LBH antibody validated for IHC [9, 10]. In agreement with our meta-analysis, LBH protein was significantly overexpressed in cancers of the gastrointestinal tract (i.e., PAAD, ESCA, COAD, READ, LIHC, STAD), the urogenital system (i.e., KIRC, PRAD, TGCT), and in head and neck (HNSC) and thyroid cancer compared to normal tissues (Fig. 2A). Conversely, LBH immunostaining was significantly decreased in solid cancers of the lung (LUSC), skin (SKCM), ovaries (OV), and in non-Hodgkin’s lymphoma (Fig. 2B). Collectively, these data indicate a widespread dysregulation of LBH expression in cancer, whereby it is overexpressed in most cancer types except for a few.

### Association between *LBH* dysregulation, tumor stage, and patient prognosis

**Fig. 3:**
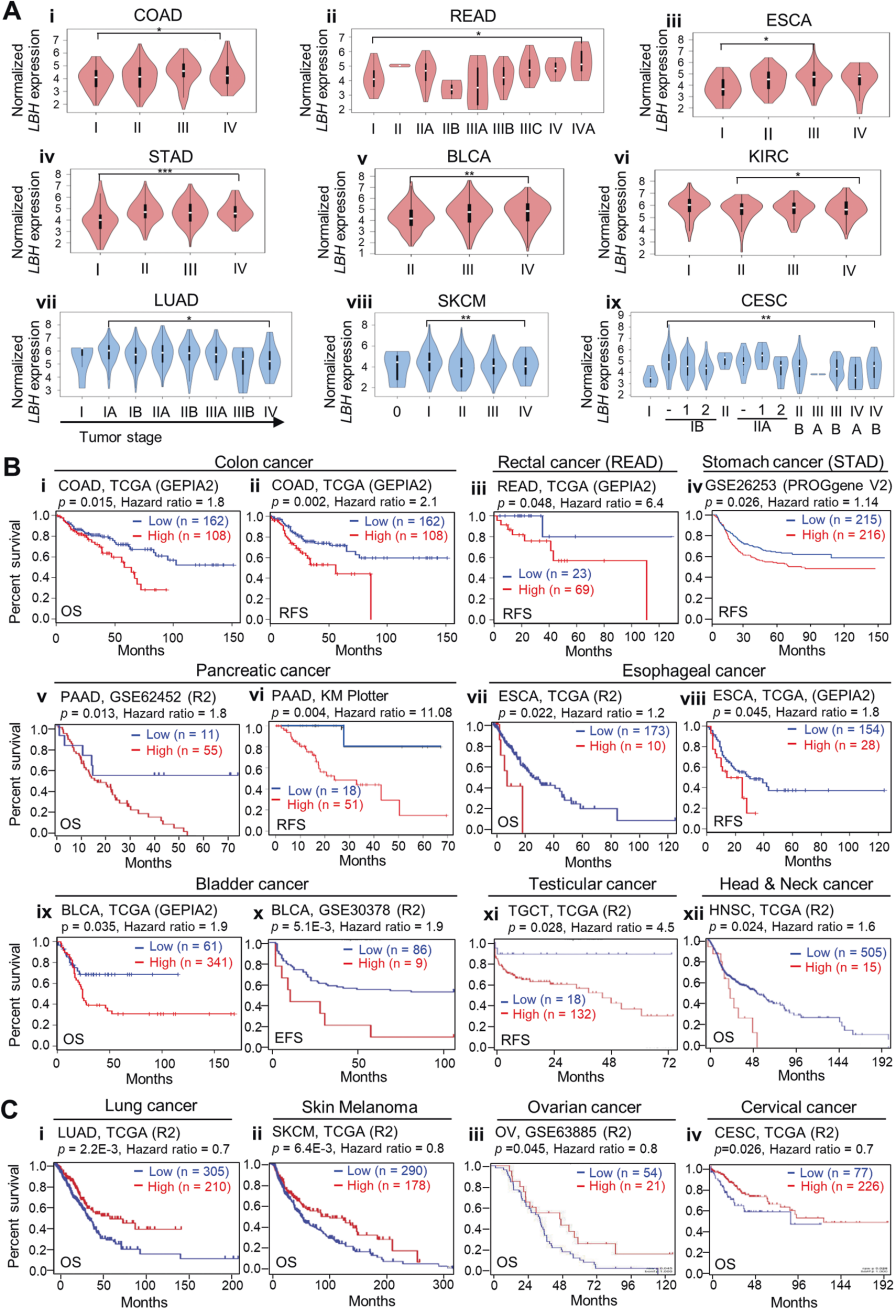
Correlation of LBH expression with tumor stage and patient survival. **A** Violin plots showing significant *LBH* expression changes across different tumor stages in multiple cancer types. (i–vi) Cancer types with increased *LBH* expression levels in the later tumor stages (III/IV) were: (i) COAD - colon adenocarcinoma, (ii) READ - rectum adenocarcinoma, (iii) ESCA - esophageal carcinoma, (iv) STAD - stomach adenocarcinoma, (v) BLCA - bladder urothelial carcinoma, and (vi) KIRC - kidney renal clear cell carcinoma. (vii–ix) Cancer types with decreased *LBH* expression in the later stages were: (vii) LUAD - lung adenocarcinoma, (viii) SKCM - skin cutaneous melanoma, and (ix) CESC Cervical squamous cell carcinoma. Violin plots were generated from GEPIA2. One-way ANOVA test: *P*-values as indicated. **B**–**C** Kaplan–Meier plots showing overall and relapse-free survival of patients with high (red) and low (blue) intra-tumoral *LBH* expressions in: **B** *LBH*-overexpressing, and **C** *LBH*-underexpressing cancer types. The TCGA-GEPIA, R2 platform (R2), and Kaplan–Meier (KM) plotter patient cohorts used, and the number (*n*) of cases with *LBH* high and low expression are indicated. Log-rank test: *P*-values (threshold *p* < 0.05) and Hazard Ratios (HR), as indicated. OS overall survival, RFS relapse-free survival, EFS event-free survival. See also Fig. S2.

To assess if dysregulation of LBH correlated with tumor progression, we examined the association of *LBH* expression with tumor stage in cancer-specific TCGA datasets (Fig. 3A). Among cancers with *LBH* overexpression, colon (COAD), rectal (READ), esophageal (ESCA), stomach (STAD), bladder (BLCA), and kidney (KIRC) showed significant *LBH* upregulation in the latter tumor stages (III and IV; *p* < 0.05; Fig. 3A.i–vi). In contrast, in cancers with *LBH* underexpression, i.e., lung (LUAD), skin (SKCM), and cervical (CESC), reduced *LBH* expression levels correlated with advanced tumor stage (III and IV; *p* < 0.05; Fig. 3A.vii–ix).

We next used several online tools, R2, Kaplan–Meier plotter, and PROGene V2, for all the available sources from Gene Expression and Omnibus (GEO) and TCGA, to analyze the correlation between *LBH* expression and disease outcome (Fig. 3B, C). Notably, high levels of *LBH* were significantly associated with reduced overall and/or relapse-free patient survival in colon-rectal cancer (Fig. 3B.i–iii; Fig. S2A.i–ii), stomach cancer (Fig. S2A.iii–iv), pancreatic cancer (Fig. 3B.v–vi; Fig. S2.v), esophageal cancer (Fig. 3B.vii–viii; Fig. S2A.vi–vii), bladder cancer (Fig. 3B.ix–x), testicular cancer (Fig. 3B.xi), head and neck cancer (Fig. 3B.xii), sarcoma (Fig. S2A.viii), glioblastoma (Fig. S2A.ix–x), and Diffuse Large B-cell lymphoma (Fig. S2A.xi–xii), suggestive of an oncogenic role.

Contrarily, in lung cancer (Fig. 3C.i; Fig. S2B.i–ii), melanoma (Fig. 3C.ii; Fig. S2B.iii–iv), ovarian (Fig. 3C.iii), and cervical cancer (Fig. 3C.iv), high *LBH* was associated with prolonged overall patient survival, suggesting a tumor suppressive role for LBH in these cancer types.

### LBH overexpression in cancer correlates with DNA hypomethylation and poor prognosis

To uncover the mechanisms underlying the dysregulated *LBH* expression in different cancer types, we analyzed genetic alterations in *LBH* using cBioPortal. We queried *LBH* gene mutations in 90,279 samples from 202 studies, covering the entire set of available cancers. The *LBH* gene was altered in only 40 queried samples, with a somatic mutation frequency of 0.04% (Fig. S3A, B), indicating that *LBH* mutations in cancer are rare. Mutated sites were concentrated in *LBH* exons 2 and 3 (Fig. S3A), which encode the majority of LBH protein (amino acids 9 to 105) [3]. Among these mutations, 28 missense and 7 truncation mutations were detected (Fig. S3A). Next, we analyzed the frequency of *LBH* gene mutations, selecting the top 20 cancer types with at least 100 patient cases and covering a total of 15,597 samples (Fig. S3B). The average gene alteration frequency was <2% (Fig. S3B), which is low compared to those of protooncogenes (i.e., *MYC*, *ERBB2*, *RAS*) that typically show sporadic gene amplification frequencies of > 20% in many cancers [36]. Thus, genetic alterations may not be the driving force of *LBH* dysregulation in cancer.

**Fig. 4:**
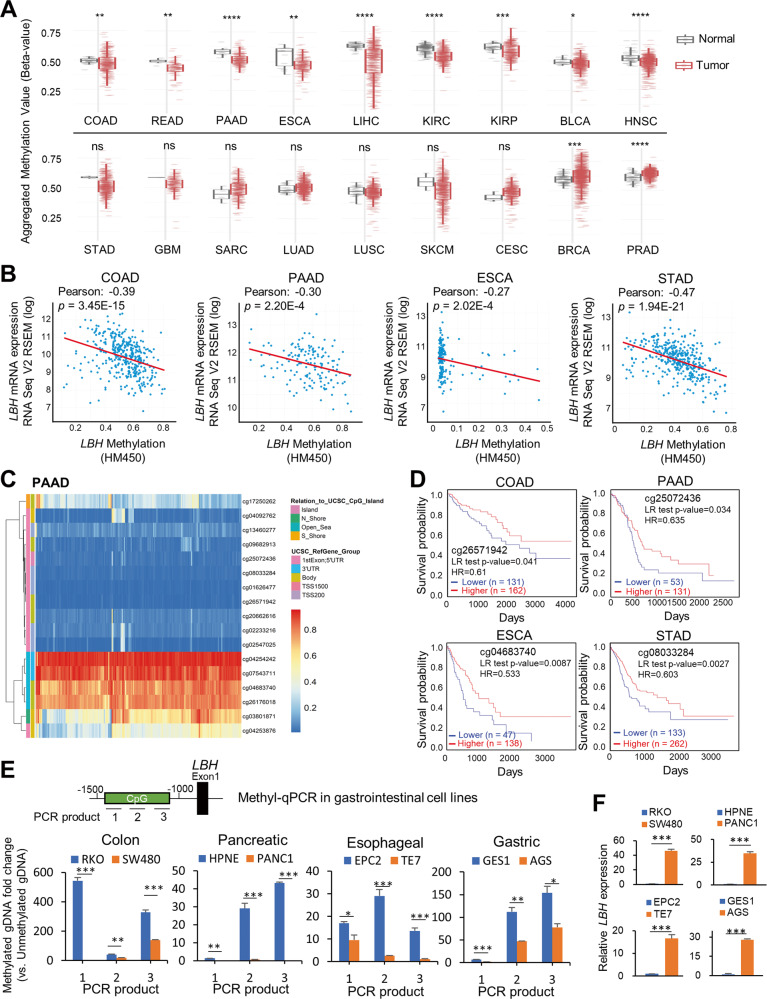
*LBH* DNA methylation status and prognostic value in cancers. **A** CpG methylation level of *LBH* in normal and primary tumor tissue among cancer types in TCGA. Box plots were generated using SMART webtool. Wilcoxon rank sum test; *P*-values: *p ≤ 0.05; **p ≤ 0.01; ***p ≤ 0.001; ****p ≤ 0.0001; ns – not significant. **B** Correlation of *LBH* mRNA expression with *LBH* DNA methylation status in COAD, PAAD, ESCA, and STAD patient datasets from TCGA via cBioportal. Pearson correlation coefficients and p-values, as indicated. **C** Heatmap of DNA methylation status at CpG islands and other sites in the *LBH* locus in pancreatic cancer (PAAD) patients from TCGA, using MethSurv. Blue signifies low, and orange equals high DNA methylation. **D** Correlation of *LBH* methylation level and the prognosis of cancer patients with PAAD, ESCA, COAD, and STAD. Kaplan–Meier survival curves were generated for selected *LBH* CpG methylation sites and survival probabilities for high (red) and low (blue) DNA methylation groups are shown. Log-rank test: *P*-values (threshold *p* < 0.05) and Hazard Ratios (HR), as indicated. See also Fig. S3 and Table S4. **E** Methylation-specific qPCR (MSP) analysis of *LBH* promoter-associated CpG islands (see Methods) in colon (SW480), pancreatic (PANC1), esophageal (TE7), and gastric (AGS) cancer cell lines compared to low-tumorigenic (RKO) or normal-derived (HPNE, EPC2, GES1) lines. Three overlapping primer sets detecting unmethylated or methylated gDNA in a CpG island −1500 to −1000 upstream of the *LBH* promoter were used. The location of the resulting PCR products is indicated. **F** qPCR validation of *LBH* mRNA expression in the same cell lines as in (**E**). Data in (**E**) and (**F**) represent mean ± SEM (*n* = 3). *P*-values: *p ≤ 0.05; **p ≤ 0.01; ***p ≤ 0.001 (Student *t*-test).

Given that DNA methylation is a key epigenetic mechanism affecting gene regulation and tumor development [37], we next analyzed association between *LBH* expression and methylation status in TCGA datasets. Notably, DNA methylation levels at the *LBH* gene locus were significantly lower in cancers with *LBH* overexpression: i.e., colon-rectal (COAD, READ; *p* < 0.01), pancreatic (PAAD; *p* < 0.0001), esophageal (ESCA; *p* < 0.01), liver (LIHC; *p* < 0.0001), kidney (KIRC, *p* < 0.0001; KIRP, *p* < 0.001), bladder (BLCA; *p* < 0.05), head and neck (HNSC; *p* < 0.0001) (Fig. 4A; top row). A trend for *LBH* hypomethylation was also observed in stomach cancer (STAD), and glioblastoma (GBM), although these differences did not reach significance (Fig. 4A-bottom row). Importantly, in colon, pancreatic, esophageal, and stomach cancer reduced *LBH* DNA methylation was significantly associated with increased *LBH* mRNA expression (Fig. 4B).

Interestingly, *LBH* underexpressing cancer types, i.e., LUAD, LUSC, SKCM, CESC, did not show significant changes in *LBH* DNA methylation status compared to normal tissues (Fig. 4A-bottom row). Cancer types with significantly increased *LBH* DNA methylation were breast (BRCA; *p* < 0.001) and prostate (PRAD; *p* < 0.0001) cancer (Fig. 4A-bottom row).

DNA methylation changes have an impact not only on gene expression, but also the prognosis of cancer patients [37]. To determine if increased *LBH* gene hypomethylation in *LBH* overexpressing cancers had prognostic value, we analyzed *LBH*-specific DNA methylation sites in the four gastrointestinal cancers above. We identified a total of 18 predicted DNA methylation sites in the *LBH* locus, whereby 11 located in CpG islands (Fig. 4C; Fig. S3C). All 18 *LBH* DNA methylation sites showed significant correlation with disease prognosis in stomach cancer, while two, three, and five DNA methylation sites in *LBH* affected overall patient survival in colon, pancreatic, and esophageal cancer, respectively (Table S4). Notably, sites in *LBH-associated* CpG islands showed lower methylation in all four cancer types (Fig. 4C; Fig. S3C), in association with poor prognosis (Fig. 4D; and Table S4). Contrarily, higher methylation levels were detected at individual sites in the *LBH* gene body (cg04683740 and cg26176018) and 3’UTR (cg04254242 and cg07543711) Table S4), consistent with reports that high DNA methylation levels in gene body correlate with increased gene expression [38, 39].

To further validate *LBH*-specific DNA hypomethylation in *LBH* overexpressing cancers, we performed methylation-specific qPCR (MSP) in normal and tumorigenic gastrointestinal cell lines (Fig. 4E). Colon (SW480), pancreatic (PANC1), esophageal (TE7), and gastric (AGS) cancer lines all showed significantly decreased levels of DNA methylation at CpG islands upstream of the *LBH* promoter compared to either low-tumorigenic (colon: RKO), or normal-derived cell lines (pancreatic: HPNE, esophageal: EPC2, gastric: GES-1) (Fig. 4E). Importantly, as observed in primary tumors, tumor lines with *LBH* DNA hypomethylation showed a drastic increase in *LBH* mRNA, as determined by qPCR (Fig. 4F). Collectively, these results identify that DNA hypomethylation, particularly at *LBH*-specific CpG islands may contribute to aberrant *LBH* overexpression in cancer.

### *LBH* overexpression correlates with the WNT and Integrin signaling pathways

To identify potential targetable signaling pathways correlating with LBH deregulation in cancer, we performed a systematic analysis of *LBH* co-expressed genes across different cancer types. Based on the observed prevalent and prognostically significant *LBH* overexpression in gastrointestinal cancers (Figs. 1–4), we started this analysis in COAD, STAD, LIHC, PAAD, and ESCA. We first queried individual cancer datasets from TCGA to identify *LBH* co-expressed genes using the R2 platform with Bonferroni correction and a *p*-value < 0.01. We then overlapped these gene clusters from at least three different cancers using PANTHER to identify common LBH-associated pathways.

**Fig. 5:**
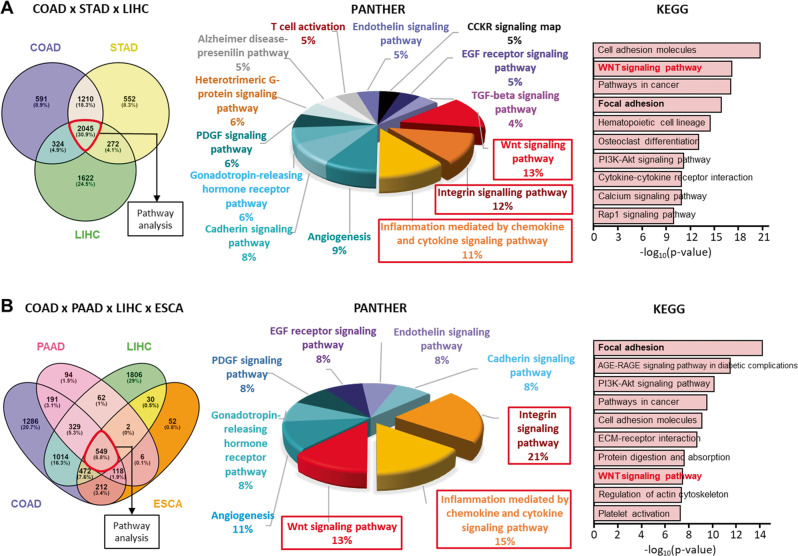
Enrichment Analysis of *LBH* co-expressed signaling pathways. **A**, **B** Venn diagram of genes positively correlated with *LBH* in the cancer types indicated (left). The identified common genes from multi-cancer analysis were used for PANTHER (middle) or KEGG (right) pathway analysis. Pathways that have at least 10 predicted common genes are listed. **A** Analysis of *LBH* positively correlated genes in three cancer types: COAD, STAD, and LIHC; or **B**, in a four-cancer combination: COAD, PAAD, LIHC, and ESCA. Note that the top three pathways are WNT, Integrin, and inflammation signaling throughout different cancer-type combinations. COAD; colon adenocarcinoma, ESCA; esophageal carcinoma, LIHC liver hepatocellular carcinoma, PAAD pancreatic adenocarcinoma, STAD stomach adenocarcinoma. See also Fig. S4 and Table S5.

We identified 2045 positively and 38 negatively LBH-associated genes common to COAD, STAD, and LIHC (Fig. 5A), and 1066 positively and 44 negatively LBH-associated when COAD, STAD, and PAAD were compared (Fig. S4A). Notably, in both these 3-cancer comparisons, as well as in 4-cancer comparisons including esophageal cancer (ESCA) (Fig. 5B; and Fig. S4B), the WNT (12–13%), Integrin (12–22%), and inflammation mediated by chemokine and cytokine (11–15%) were the top three signaling pathways among positively *LBH*-correlated genes. Other common pathways were: Angiogenesis (8–9%), Cadherin signaling (7–9%), Gonadotropin-releasing hormone receptor pathway (7–10%), PDGF (6–8%) and EGF receptor (5–7%) signaling, Endothelin signaling (5–8%), and in some 3-cancer comparisons also Heterotrimeric G-protein signaling (5%), CCKR signaling (5%), the Alzheimer disease-presenilin pathway (4-5%), TGFβ (4-5%) and VEGF (4%) signaling, as well as T cell activation (4%) (Fig. 5; and Fig. S4). Independent KEGG analysis confirmed the WNT and Integrin (= Focal Adhesion) signaling pathways as top *LBH*-coexpressed pan-cancer gene signatures (Fig. 5; and Fig. S4). There were no reliant gene signatures from negatively *LBH*-correlated gene clusters due to low gene numbers (data not shown).

We extended our analysis to a total of 10 *LBH* overexpressing cancer types to examine the consistency and universality of *LBH-*associated signaling pathways. Encouragingly, highly consistent results were obtained in kidney/KIRC, bladder/BLCA, prostate/PRAD, and brain/GBM cancers, with the WNT and Integrin signaling pathways being consistently enriched (Supplementary Table S5).

Notably, in colon, stomach, and kidney cancer, WNT was the top enriched pathway among *LBH-*coexpressed genes (Supplementary Table S5), consistent with *LBH* being a direct WNT/β-catenin target gene [13]. Moreover, WNT was the second most enriched LBH-associated pathway in liver, pancreatic, rectal cancer; the third most enriched in esophageal (ESCA) and bladder (BLCA) cancer, and the fourth in prostate cancer (PRAD) (Supplementary Table S5). Even in a cancer, as divergent as glioblastoma, WNT was second most enriched (Supplementary Table S5), indicating a high degree of correlation between *LBH* and WNT signaling activity in cancer.

We also analyzed the pathways associated with *LBH* underexpression in lung and skin cancer. No common enriched pathways could be detected due to the low number of overlapping genes (78 genes). Nonetheless, individual pathway analysis showed that in LUAD, the top five pathways inversely correlated with *LBH* expression were the ubiquitin-proteasome pathway (14%), de novo purine and/or pyrimidine biosynthesis (13% and 8%, respectively), DNA replication (13%), and Parkinson disease (10%) (Fig. S5). The p53 pathway (6%) was also enriched, consistent with a tumor suppressive role of LBH in lung cancer [22]. In SKCM, de novo purine biosynthesis was the top enriched pathway (17%), followed by Huntington disease (17%), TGFß signaling (14%), the Alzheimer disease-presenilin pathway (11%), and Adrenaline and noradrenaline biosynthesis (11%) (Fig. S5). These results reveal possible new functions of LBH in regulating RNA/DNA synthesis, protein degradation, and in neurodegeneration.

### Prognostic interactions between LBH and the WNT and Integrin signaling pathways

**Fig. 6:**
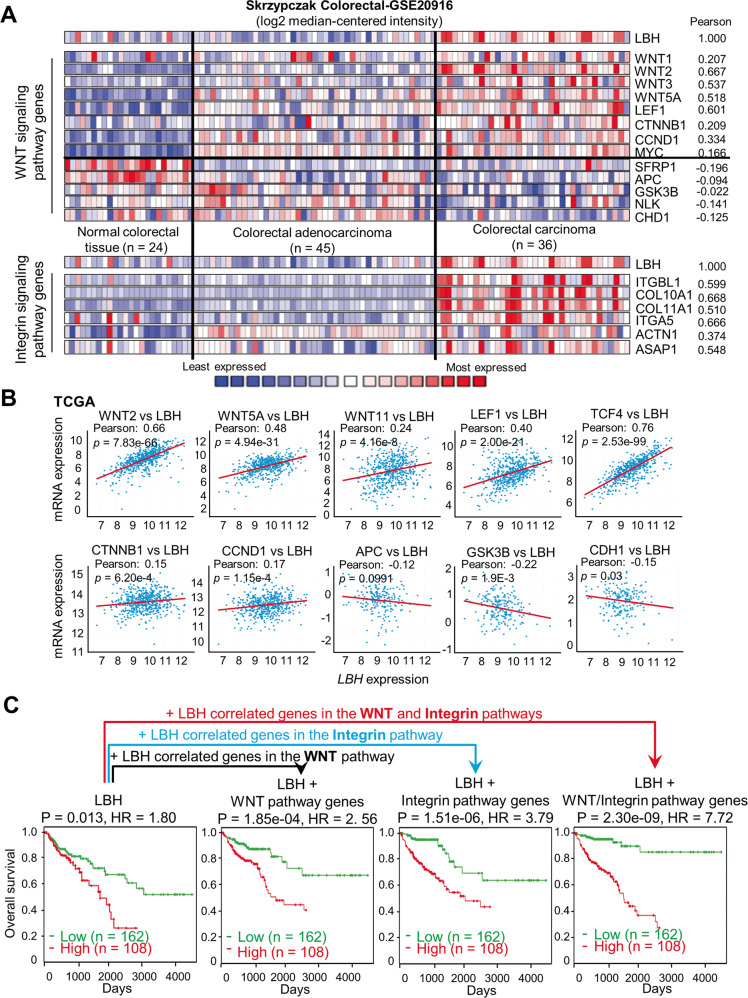
*LBH* overexpression in colon cancer correlates with WNT-Integrin signaling genes, predicting poor outcome. **A**, **B** Co-expression and correlation analysis of LBH with WNT/Integrin pathway genes. **A** Heatmaps of *LBH* and correlated genes in a representative colorectal cancer dataset from Oncomine (Skrzypczak_Colorectal-GSE20916). Blue signifies low, and red equals high mRNA expression. Pearson correlation coefficients between *LBH* and indicated genes are listed. **B** Dot plots showing correlations between *LBH* expression and expression of key WNT pathway genes in colon cancer based on TCGA data. Pearson correlation coefficients (R-values) and P-values, as indicated. See also Figs. S6, S7, S8. **C** Kaplan−Meier plot of overall survival in colon cancer patients (*n* = 270) stratified by the LBH/WNT/Integrin signatures. Multivariate Cox regression analysis of COAD patient data from TCGA. Log-rank test: *P*-values and Hazard Ratios (HR), as indicated. See also Table S6.

Since aberrant *LBH* overexpression showed the highest pan-cancer correlation with the WNT and Integrin signaling pathways, we explored the clinical and prognostic significance of these associations in more detail. Heatmap analysis in colorectal, stomach, and pancreas cancer patient datasets showed a consistent overlap between *LBH* overexpression and WNT signaling genes associated with pathway activation, i.e., *WNT2, WNT5A, WNT11, LEF1, TCF4* or *TCF7, CCTNB1*, *CCND1, JUN, DKK3* (Fig. 6A, B; and Figs. S6, S7, S8A). In contrast, *LBH* was inversely correlated with pathway genes associated with WNT signaling repression, i.e., *APC*, *GSK3B*, and *CDH1*. Similar significant correlations between *LBH* and key WNT pathway genes were observed in esophageal, liver, and brain cancers (Fig. S8B–D).

The Integrin pathway genes most strongly associated with *LBH* were: *ITGBL1, ACTN1*, and collagen genes, although those differed in the various gastrointestinal cancers (*COL10A1, COL11A1* in the Skrzypczak_Colorectal-GSE20916; *COL4A1, COL4A2* in both the Badea_Pancreas-GSE15471 and Chen_Gastric [40] datasets) (Fig. 6A; and Figs. S6A, S7A).

We next performed multivariate Cox analysis to combine *LBH* expression and WNT/Integrin gene signatures with time of overall patient survival in the COAD, STAD, and PAAD cohorts from TCGA into an integrated risk score model. While expression of *LBH*, WNT, or Integrin pathway genes alone showed Hazard ratios of 1.80 (*p* = 0.015), 2.54 (*p* = 1.73E-04) or 3.77 (1.42E−06), respectively at a Confidence Interval of 95%, combinations of *LBH* with WNT or Integrin, or all three gene clusters combined increased predictive power linearly for the overall survival of patients to an HR of 7.72 (*p* = 2.30E−09) in COAD, an HR of 7.41 (*p* = 6.61E−14) in PAAD, and an HR of 5.95 (*p* = 1.62E−08) in STAD (Fig. 6C; and Table S6). Collectively, these data demonstrate that a combined *LBH*-WNT-Integrin gene signature reliably stratifies colon, pancreatic, and stomach cancer patients into high- and low-risk groups for cancer survival.

### Clinical association between LBH and WNT hyperactivation in gastrointestinal cancers

**Fig. 7:**
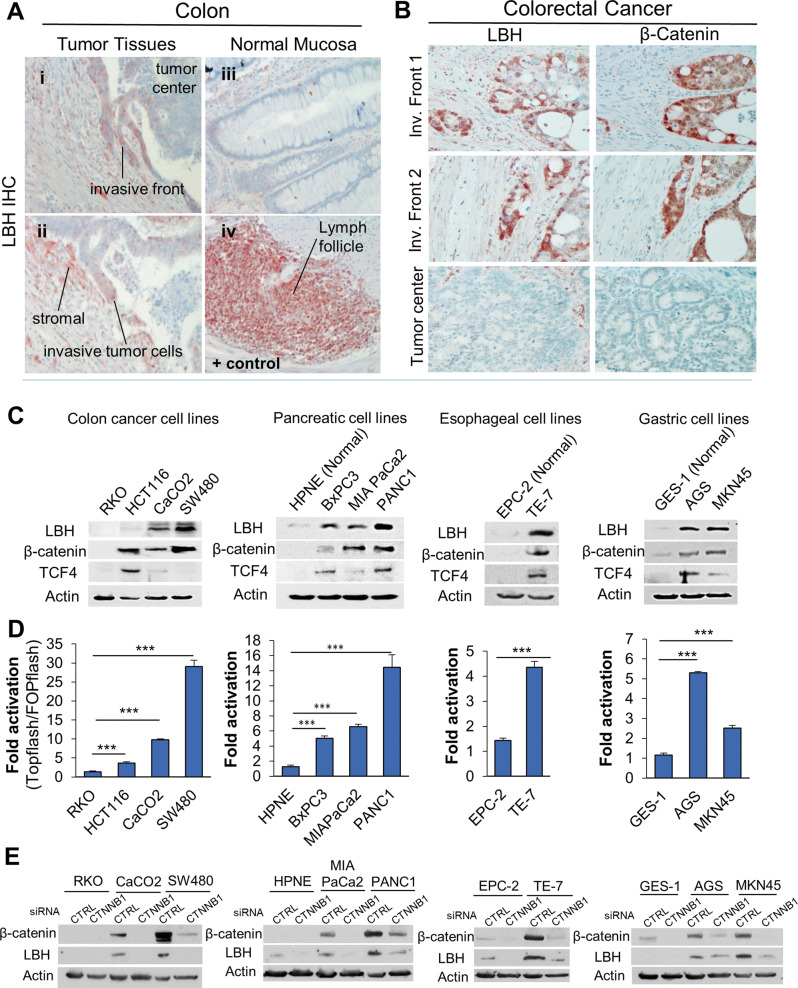
Clinical correlation between LBH protein expression and WNT hyperactivation in gastrointestinal cancers. Immunohistochemical (IHC) analysis of LBH protein expression (red/brown) in invasive colorectal carcinoma (CRC) specimen with validated anti-LBH antibody [9, 10]. **A** LBH is overexpressed with a predominant nuclear pattern in tumor cells at the invasive edge of CRC tumors (i–ii), compared to adjacent normal gut mucosa (iii). LBH-specific immunopositivity was also detected in tumor-associated stroma (**i-ii**). Gut-associated lymph follicles served as positive (+) control for LBH immunostaining (iv). **B** Representative IHC images of serial paraffin sections immunostained with LBH or ß-catenin antibodies. LBH expression in CRC cells at the invasive tumor front (Inv. Front) correlates with nuclear β-catenin staining, indicative of active WNT signaling. Note, that neither LBH nor β-catenin was expressed in the tumor center. **C** Western blot analysis of LBH, β-catenin, and TCF4 in CRC cell lines with no (RKO), low (HCT116), and high endogenous (Caco2, SW480) WNT activity, and in normal and tumorigenic pancreatic, esophageal, and gastric cell lines, as indicated. β-Actin (loading control). **D** Transcriptional luciferase reporter assays in the same cell lines above (**C**), using WNT responsive TOPFlash relative to FOPFlash reporter activity. Data are means ± SEM from three experiments performed in duplicates. *P*-values: *p ≤ 0.05; **p ≤ 0.01; ***p ≤ 0.001 (one-way ANOVA). **E** Western blot analysis of LBH protein expression in selected normal and tumorigenic GI cell lines above (**C**) 72 h after transient transfection of cells with β-catenin (*CTNNB1)* siRNA or scramble control (CTRL) siRNA.

The WNT/ß-catenin signaling pathway is abnormally activated in a variety of cancers and represents a key molecular target for cancer stem cell (CSC)-targeted anti-cancer therapy [41, 42]. WNT inhibitors are in clinical trials [42, 43]. However, reliable biomarkers to detect WNT signaling activity in tumor specimens are lacking. Aberrant WNT hyperactivation (through stabilizing mutations in ß-catenin (*CTNNB1*), or in the adenomatous polyposis coli, *APC*, gene) is particularly common in colorectal cancer (CRC = COAD + READ), and is a major driver of CRC development [44]. Therefore, to validate the clinical association between LBH overexpression and WNT pathway activation in cancer, we performed IHC analysis in a cohort of CRC patients (Fig. 7A, B). LBH-positive immunostaining was detected in 14 of 18 different CRC patient samples analyzed (Fig. 7A.i–ii; Fig. 7B; and data not shown), confirming LBH overexpression in colon cancer (Fig. 1; Fig. S1A). LBH was predominantly expressed in CRC cells at the tumor invasive front, and in tumor-associated stroma (Fig. 7A.i–ii). In contrast, LBH was not expressed in normal colon mucosa of adjacent tissue (Fig. 7A.iii), indicating it is a tumor-specific marker. Importantly, staining of adjacent tumor sections with antibodies to β-catenin revealed that LBH was expressed in a subset of invasive CRC cells with nuclear β-catenin expression (Fig. 7B), which is a hallmark of WNT activation [45]. The overlap between LBH and nuclear β-catenin positive cases was 100%, as only the 14 tumors with positive LBH immunostaining also showed nuclear β-catenin immunostaining (Fig. 7B; and data not shown). Notably, the positive regions for LBH and nuclear β-catenin within individual cases were highly overlapping (mainly invasive tumor regions) and neither LBH, nor nuclear β-catenin were detected in differentiated tumor centers (Fig. 7B), consistent with previous studies showing that WNT signaling activity is concentrated in invasive CRC cells [45].

We next analyzed LBH protein expression and WNT pathway activation by Western blot analysis and TOPFLASH reporter assays in CRC cell lines with no (RKO), low (HCT116 – CTNN1mut), and high (CaCO_2_, SW480 – APCmut) WNT activation (Fig. 7C, D). We included in these analyses cell lines models for pancreatic (BxPC3, MIAPaCa2, PANC1), esophageal (TE7), and gastric (AGS, MKN45) cancer, as these cancer types also showed prognostically significant association between *LBH* expression and WNT gene signatures (Figs. S6–8; and Table S6). While low-tumorigenic RKO, and normal-derived pancreatic (HPNE), esophageal (EPC2), and gastric (GES-1) cell lines had no or very low levels of LBH protein, LBH was markedly increased in all cancer lines analyzed (Fig. 7C). Notably, LBH upregulation in CRC, PAAD, ESCA, and STAD cancer lines coincided with both, increased β-catenin and TCF4 protein expression (Fig. 7C), as well as increased TOPFlash reporter activity, a measurement of β-catenin/TCF transcriptional activity (Fig. 7D). Significantly, knockdown of β-catenin profoundly reduced the elevated LBH expression in gastrointestinal tumor lines (Fig. 7E), uncovering that LBH overexpression in gastrointestinal cancers is WNT-dependent.

These experimental data, together with our present, and previous meta-analyses [13, 46], indicate that LBH may be a universal biomarker to detect WNT hyperactivation in cancer.

## Discussion

Here we performed the first comparative analysis of *LBH* expression in >20 different cancer types, using meta-analysis of published gene expression data, TMA analysis, and experimental validation in cancer cell lines, revealing overexpression of LBH in most cancer types except for a few.

**Fig. 8:**
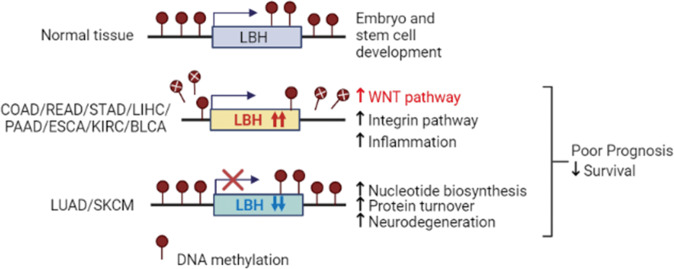
Model summarizing the multi-cancer *LBH* meta-analysis. In normal tissues, LBH regulates stem cells in embryonic development and tissue homeostasis. In gastrointestinal (COAD, READ, STAD, LIHC, PAAD), urological (KIRC, BLCA), and other cancers, the *LBH* gene locus is hypomethylated leading to increased *LBH* expression, in association with WNT/Integrin and inflammatory signaling pathways. In lung (LUAD) and skin (SKCM) cancer, *LBH* is downregulated, without DNA methylation changes, correlating with nucleotide synthesis, protein turnover, and neurodegeneration gene signatures, suggesting novel mechanisms of LBH regulation and function in carcinogenesis.

We confirmed the overexpression of *LBH* in glioblastoma, stomach, and liver cancers, and its underexpression in lung cancer, consistent with previous reports [15–19, 22], expanding prognostic and molecular insight into *LBH* dysregulation in these cancers. Importantly, we newly identified aberrant *LBH* overexpression in pancreatic, esophageal, colon, rectal, bladder, kidney, prostate, testicular, head & neck cancers, and in sarcoma. High intra-tumoral LBH expression in these cancers was significantly associated with reduced patient survival, and/or advanced tumor grade, implying novel oncogenic LBH functions. In contrast, we identified *LBH* underexpression in melanoma, ovarian, cervical, and uterine-endometrial cancers, where it was associated with good prognosis, suggestive of tumor suppressive roles. Thus, *LBH* is prevalently overexpressed in gastrointestinal, urological, connective tissue, and brain cancers, but appears downregulated in cancers arising from surface ectoderm, i.e., lung and skin, and in certain gynecological malignancies, revealing a unique pattern for LBH dysregulation in solid tumors (Fig. 8).

Analysis of *LBH* expression in hematopoietic malignancies, for which data had been lacking, revealed that in blood cancers *LBH* is both over- and under-expressed in a subtype-specific manner. While *LBH* was overexpressed in aggressive pediatric leukemia (i.e., B-ALL), and in the most common lymphoma subtype, Diffuse large B-cell lymphoma, correlating with reduced patient survival, it was under-expressed in late-onset adult leukemia/lymphomas, and in myeloma.

Several discrepancies between our data and previously published reports are noted. First, both Oncomine and TCGA whole patient cohorts show downregulation of *LBH* mRNA in invasive breast cancers (BRCA) compared to normal breast tissue, seemingly in contradiction with its reported oncogenic roles in breast carcinogenesis [13, 14, 46]. This is likely due to that LBH is not expressed in majority of hormone receptor-positive, luminal-type breast cancers [13, 46], which account for 70–80% of breast cancers. However, LBH is overexpressed in worst prognosis basal-like breast cancers [13, 46], the most aggressive and lethal tumor subtype, affecting 16% of breast cancer patients [47]. As a result, by analyzing whole patient cohorts the subtype-specific *LBH* overexpression in these difficult-to-treat breast cancers may be diluted, leading to inconsistent results.

Second, LBH has first been shown to have tumor suppressive activity in cell line models of nasopharyngeal cancer (NPC), a rare head and neck cancer arising from nose-throat epithelium [21]. In contrast, our meta-analysis shows consistent *LBH* overexpression, correlating with reduced patient survival, in head and neck squamous cell carcinoma (HNSC), the most common head and neck cancer type originating from mucous epithelium of the oral cavity, pharynx, and larynx. NPC to a large degree is caused by Epstein-Barr virus (EBV) infection [21], and, thus, may have a different etiology than HNSC. Indeed, the EBV-encoded LMP1 protein has been shown to downregulate *LBH* expression in nasopharyngeal epithelial cells [21]. Interestingly, although the IHC study by Liu et al. clearly showed LBH downregulation in majority of NPC tumors, positive LBH immunostaining was detected in squamous cell NPC [21]. Thus, even in cancers with reported LBH underexpression there appear to be individual subtypes with LBH overexpression. Future studies are needed to address the mechanisms underlying the potential dual roles of LBH within certain cancer types.

Third, we found *LBH* was significantly overexpressed in prostate cancer (PRAD) in both the TCGA and Oncomine patient cohorts (>1.6-fold; *p* < 0.003) that together encompass more than 500 primary prostate cancers, and in prostate cancer tissues by IHC. This is in discrepancy with a published study, reporting LBH is downregulated in PRAD (based on a limited number of clinical samples), and to have tumor suppressive effects when overexpressed as LBH-GFP fusion protein in PC3M cells [48]. However, in agreement with our data, LBH protein is overexpressed in most prostate cancer cell lines [48, 49]. Moreover, recent LBH siRNA knockdown studies in PC3 and LnCap PRAD cells indicate it has pro-oncogenic/invasive activity [49]. Thus, our results provide vital new insight into LBH expression and function in prostate cancer, supportive of an oncogenic role.

Increased methylation of tumor suppressor genes, or decreased methylation of oncogenes can promote tumorigenesis [50, 51]. We found that DNA methylation levels at the *LBH* locus were significantly lower in *LBH* overexpressing cancers than in normal tissues. Notably, in colon, pancreatic, esophageal, and stomach cancer DNA hypomethylation of CpG islands in the *LBH* locus was associated significantly with increased *LBH* mRNA expression, as validated in cell line models, and with poor patient survival. Thus, epigenetic mechanisms facilitating DNA hypomethylation may play an important role in aberrant LBH activation in cancer (Fig. 8). Future studies should address which DNA methyltransferases may regulate this process.

In contrast, cancers with prominent *LBH* underexpression, e.g., lung, melanoma, showed no significant differences in *LBH* DNA methylation status, indicating other mechanisms are involved in *LBH* downregulation in cancer (Fig. 8). This was unexpected, as loss of *LBH* expression in the autoimmune disease, rheumatoid arthritis (RA), has been shown to involve DNA hypermethylation of an *LBH*-specific enhancer region that is associated with increased RA risk [52]. Collectively, our data point to disease-specific differences in the *LBH* DNA methylation patterns.

Genes in the same mechanistic network tend to be co-expressed and synergistically co-regulated. We previously reported that *LBH* overexpression in aggressive basal-like breast cancers is associated with WNT pathway genes [13]. Other studies have shown that *LBH* is co-expressed with the Focal Adhesion (FAK)/Integrin signaling pathway in gastric cancer [16, 17], and can both positively and negatively regulate Integrin expression [16, 22]. However, whether these associations occur in other cancer types was not known. Our pathway analysis in >10 different cancer types revealed a universal, positive association between *LBH* overexpression with both the WNT and Integrin/Focal Adhesion signaling pathways (Fig. 8). Notably, *LBH-*WNT-Integrin co-expression gene signatures showed high significance in predicting poor survival in patients with colorectal, stomach, esophageal, and pancreatic cancer. Integrins are the main transmembrane receptors regulating cell adhesion, tumor cell-extracellular matrix (ECM) interactions, and activate specific signaling pathways, which enhance tumor cell migration, invasion, proliferation, and survival [53]. Different modes of actions for integrins to activate WNT signaling have been demonstrated [54]. Conversely, WNT signaling can activate Integrin/Focal Adhesion signaling [55, 56]. Given that *LBH* is a direct WNT/β-catenin target gene in epithelial development and breast cancer [13], and has been shown to increase FAK-PI3K-AKT signaling in stomach cancer models [16], it is likely that LBH is involved in crosstalk between these two pathways. Importantly, IHC validation in colorectal patient samples revealed that LBH is specifically expressed in tumor cells with WNT signaling activity at the invasive front which engage directly with extracellular matrix to invade adjacent tissue. Future studies are needed to dissect the precise role of LBH in WNT-Integrin-mediated tumor cell invasion.

Other notable pathways positively associated with LBH were inflammation cytokine-chemokine receptor signaling, consistent with a role of LBH in immunity [12] (Fig. 8); and angiogenesis/VEGF signaling, consistent with studies in GBM models showing that LBH induces VEGF-MEK-ERK signaling [18]. Moreover, our analysis uncovered PDGF and EGF receptor signaling as LBH-associated pathways. Both are key molecular targets in anti-cancer therapy [57]. Interestingly, *LBH*, which is also a TGFβ/SMAD3 target gene [58], showed both positive and negative correlations with TGFβ signaling gene signatures. This may be due to that *LBH* induction by TGFβ is context-specific [58]. *LBH* underexpression in cancer was further associated with de novo purine/pyrimidine synthesis, DNA replication, protein ubiquitin proteasome pathway, and neurodegenerative diseases characterized by protein defects (i.e., Parkinson, Huntington, Alzheimer disease), suggesting novel functions of LBH in RNA/DNA synthesis, protein turnover, and neurodegeneration (Fig. 8).

Collectively, our multi-cancer analysis newly identified *LBH* overexpression in colon, rectal, pancreatic, esophageal, bladder, kidney, prostate, testicular, head & neck cancer, sarcoma, and in aggressive leukemia and lymphoma subtypes, revealing DNA hypomethylation as a possible mechanism of aberrant LBH activation. Contrarily, *LBH* is underexpressed, next to lung cancer, in melanoma, ovarian, uterine, and cervical cancers. Importantly, this study establishes LBH as a putative pan-cancer biomarker for detecting WNT signaling activity in clinical specimen. As WNT targeted drugs are in clinical trials, identification of LBH as a marker that could stratify patients for WNT-targeted anti-cancer therapy is of high clinical significance.

## Supplementary information

Supplemental Material

## Data Availability

All data generated or analyzed during this study can be found within the published article and its supplementary files.
