# Supplementary material for: Multi-cancer analysis reveals universal association of oncogenic LBH expression with DNA hypomethylation and WNT-Integrin signaling pathways
Source: Cancer Gene Ther. 2023 Jun 2;30(9):1234–48. doi: 10.1038/s41417-023-00633-y (PMC10501907; doi:10.1038/s41417-023-00633-y)
Supplement: Supplementary file 1 — Supplemental Material [file 41417_2023_633_MOESM1_ESM.pdf]

SUPPLEMENTARY INFORMATION

SUPPLEMENTARY FIGURES

Supplementary Figure S1

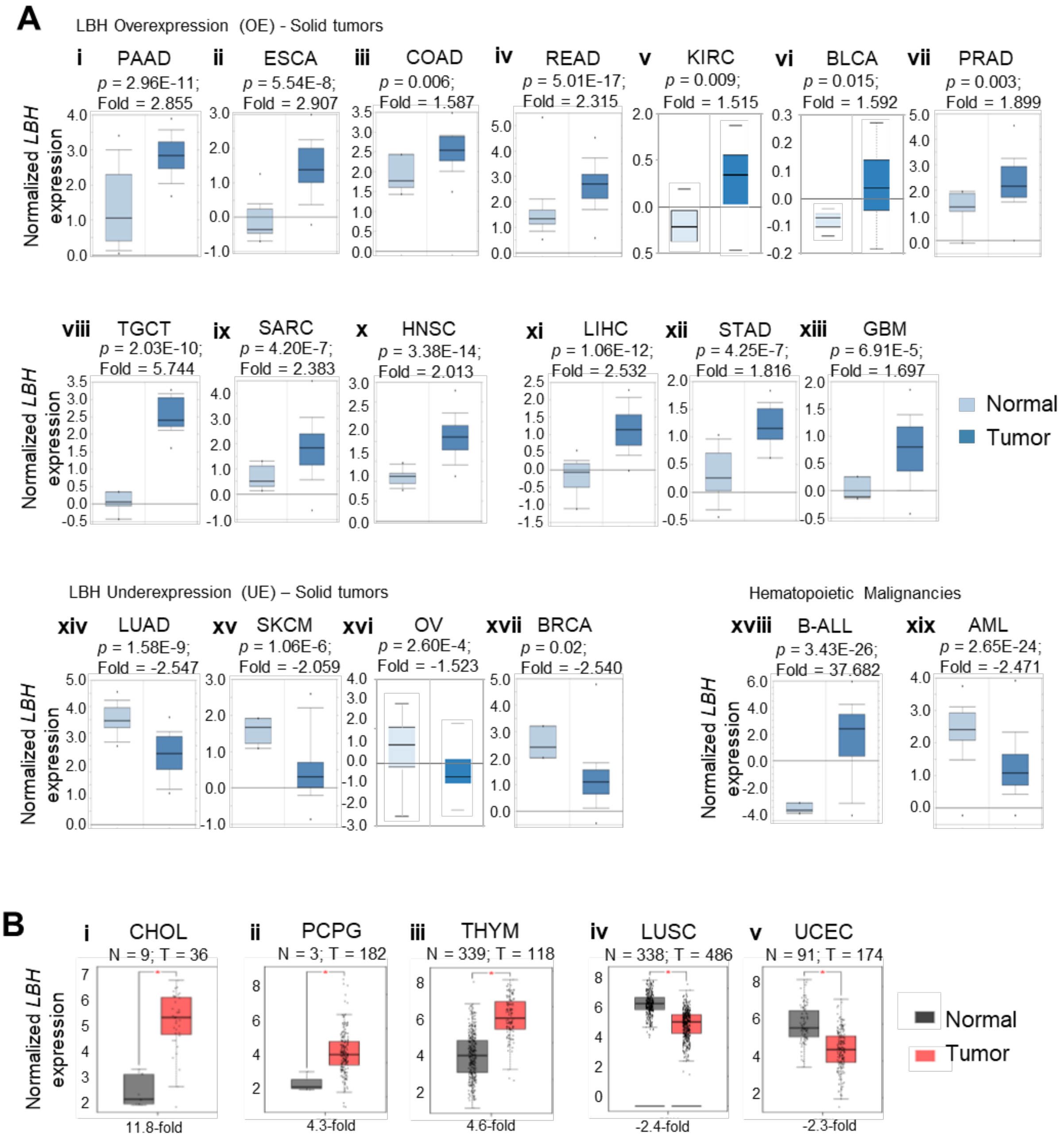

Supplementary Figure S1: *LBH* expression levels in different cancer types. A. Box plots showing differential *LBH* mRNA expression in tumor (dark blue) versus normal tissues (light blue). Cancer type-specific data was retrieved from Oncomine. Fold changes with *p*-values as indicated. (**i-xiii**) Solid tumors with *LBH* overexpression: PAAD; pancreatic adenocarcinoma, ESCA; Esophageal carcinoma, COAD; Colon adenocarcinoma, READ; Rectum adenocarcinoma, KIRC; Kidney renal clear cell carcinoma, BLCA; Bladder Urothelial Carcinoma, PRAD; Prostate adenocarcinoma, TGCT; Testicular Germ Cell Tumors, SARC; Sarcoma, HNSC; Head & Neck squamous cell carcinoma, LIHC; Liver hepatocellular carcinoma, STAD; Stomach adenocarcinoma, GBM; Glioblastoma multiforme. (**xiv-xvii)** Solid tumors with *LBH* underexpression: LUAD; Lung adenocarcinoma, SKCM; Skin Cutaneous Melanoma, OV; Ovarian serous cystadenocarcinoma, BRCA; Breast invasive carcinoma. (**xviii-xix)** Hematopoietic malignancies with *LBH* deregulation: B-ALL; B-cell acute lymphoblastic leukemia, AML; Acute Myeloid Leukemia. **B.** Box plots showing additional solid cancer types in TCGA with differential *LBH* expression in tumor (red) versus normal (grey) tissues. (i) CHOL; colon cholangial carcinoma, (ii) PCPG; pheochromocytoma and paraganglioma, (iii) THYM; thymoma, (iv) LUSC; lung squamous cell carcinoma, and (v) UCEC; uterine corpus endometrial carcinoma. The threshold was set at *p*-value = 0.05. The number of normal (N) and tumor (T) tissues is indicated for each cancer type. Data was extracted from the TCGA and GTEx databases using GEPIA2.

Supplementary Figure S2

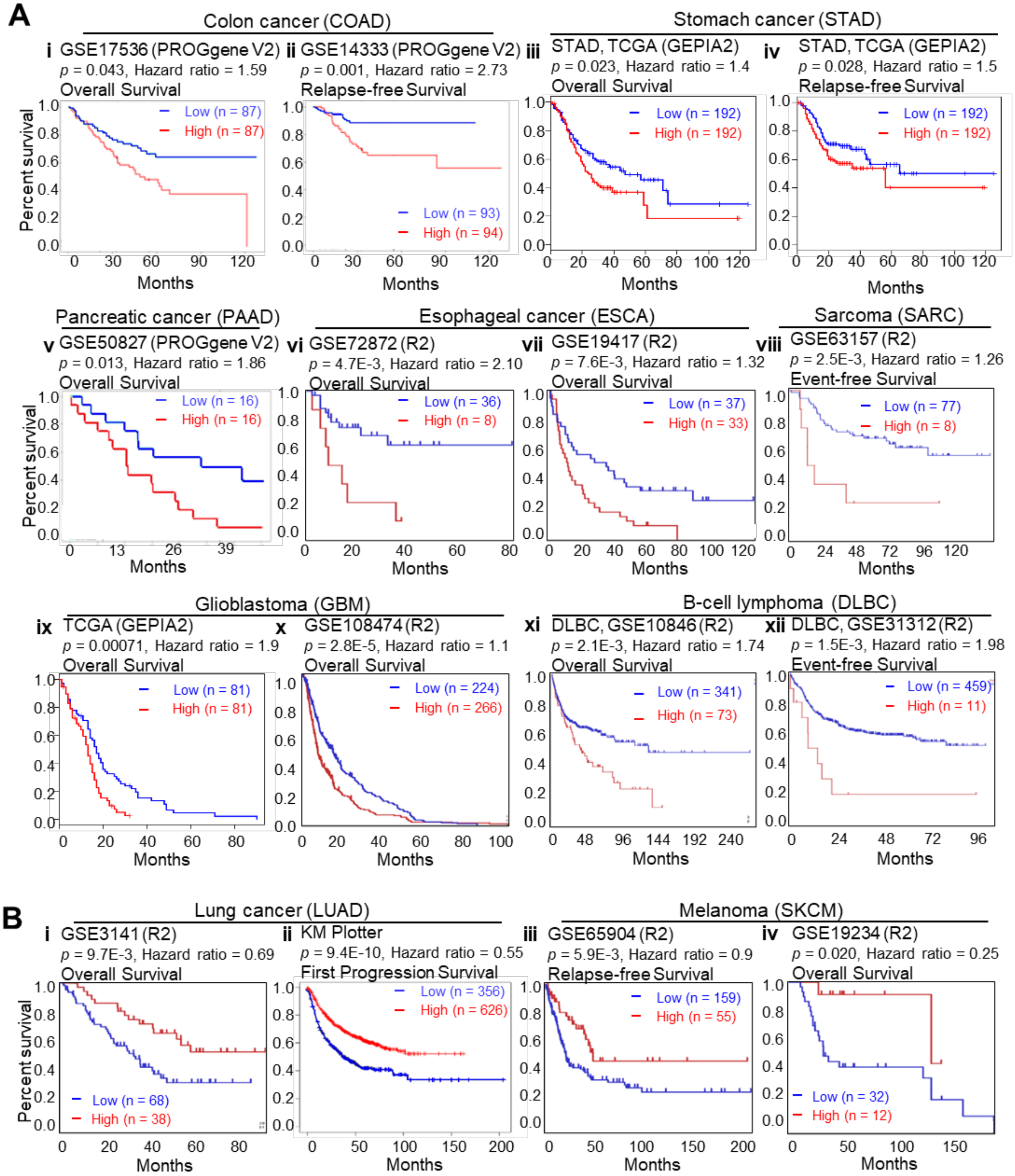

Supplementary Figure S2: Correlation of *LBH* expression with patient survival. Kaplan–Meier analysis comparing patients with high (red) and low (blue or black) *LBH* expression. **A.** High *LBH* expression correlated with poor prognosis in *LBH* overexpressing cancer types, including colon (i, ii), stomach (iii, iv), pancreatic (v), esophageal (vi, vii), sarcoma (viii), glioblastoma (ix, x), and diffuse large B-cell lymphoma (xi, xii). **B.** High *LBH* expression correlated with good prognosis in cancer types with *LBH* underexpression, i.e., lung cancer (i, ii), and melanoma (iii, iv). Log-rank test; p-value threshold < 0.05. COAD; colon adenocarcinoma, STAD; stomach adenocarcinoma, PAAD; pancreatic adenocarcinoma, ESCA; esophageal carcinoma, SARC; sarcoma, GBM; glioblastoma multiforme, DLBC; Diffuse Large B-cell lymphoma, LUAD; lung adenocarcinoma, SKCM; skin cutaneous melanoma.

Supplementary Figure S3

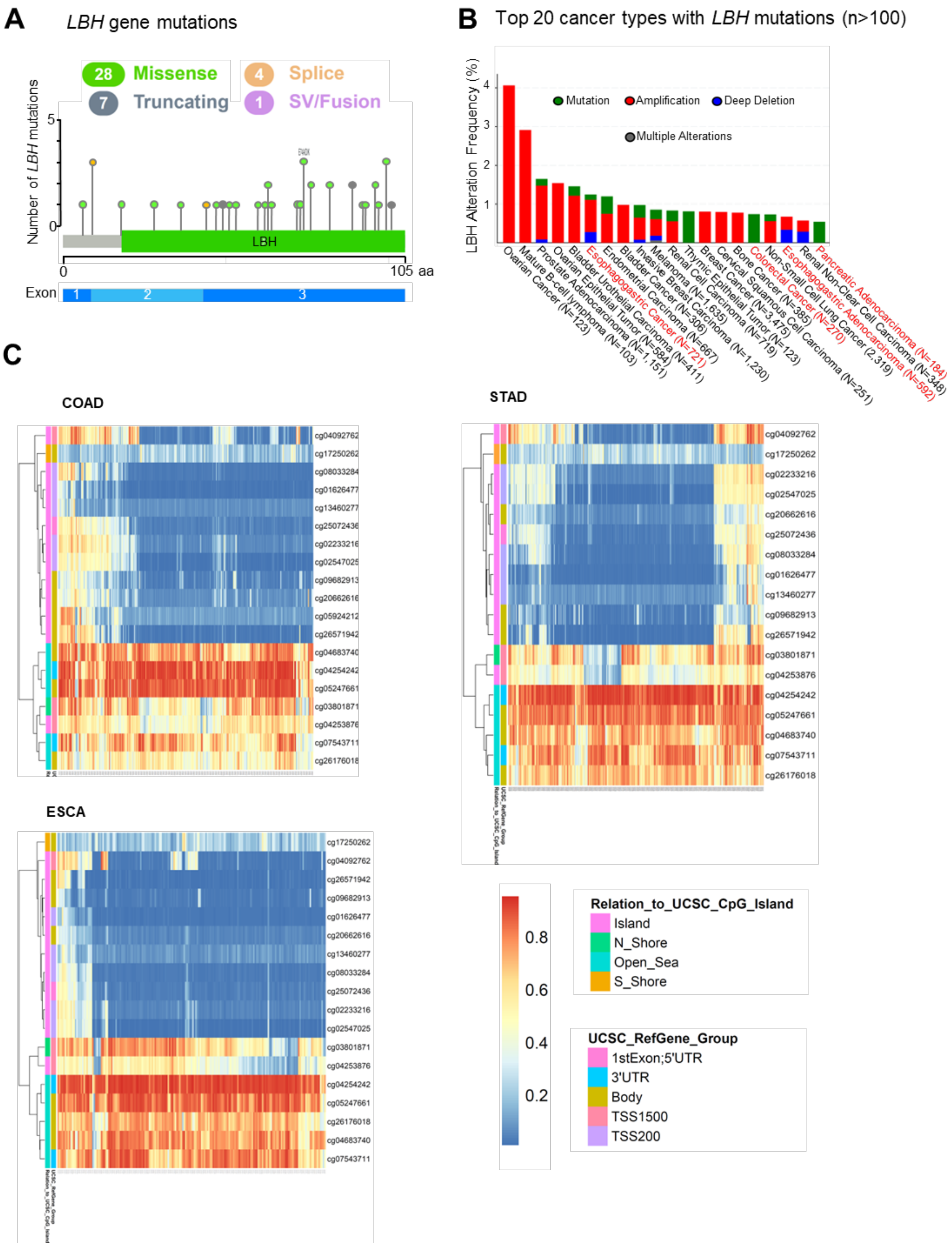

Supplementary Figure S3: Gene alteration and DNA methylation analyses for *LBH* using cBioportal. A. Forty alterations in the *LBH* gene locus were found comparing 90,279 cancer samples from 202 studies. **B.** The top 20 cancer types with *LBH* mutations. Datasets that have a sample size lower than 100 were excluded. **C.** The heatmaps of DNA methylation status at CpG sites in and around the *LBH* gene locus in COAD (n=275), ESCA (n=182), and STAD (n=408) patients. Blue signifies low, and orange equals high DNA methylation. Heatmaps were generated using the MethSurv database and patient data sets from TCGA. COAD; colon adenocarcinoma, ESCA; esophageal carcinoma, STAD; stomach adenocarcinoma.

Supplementary Figure S4

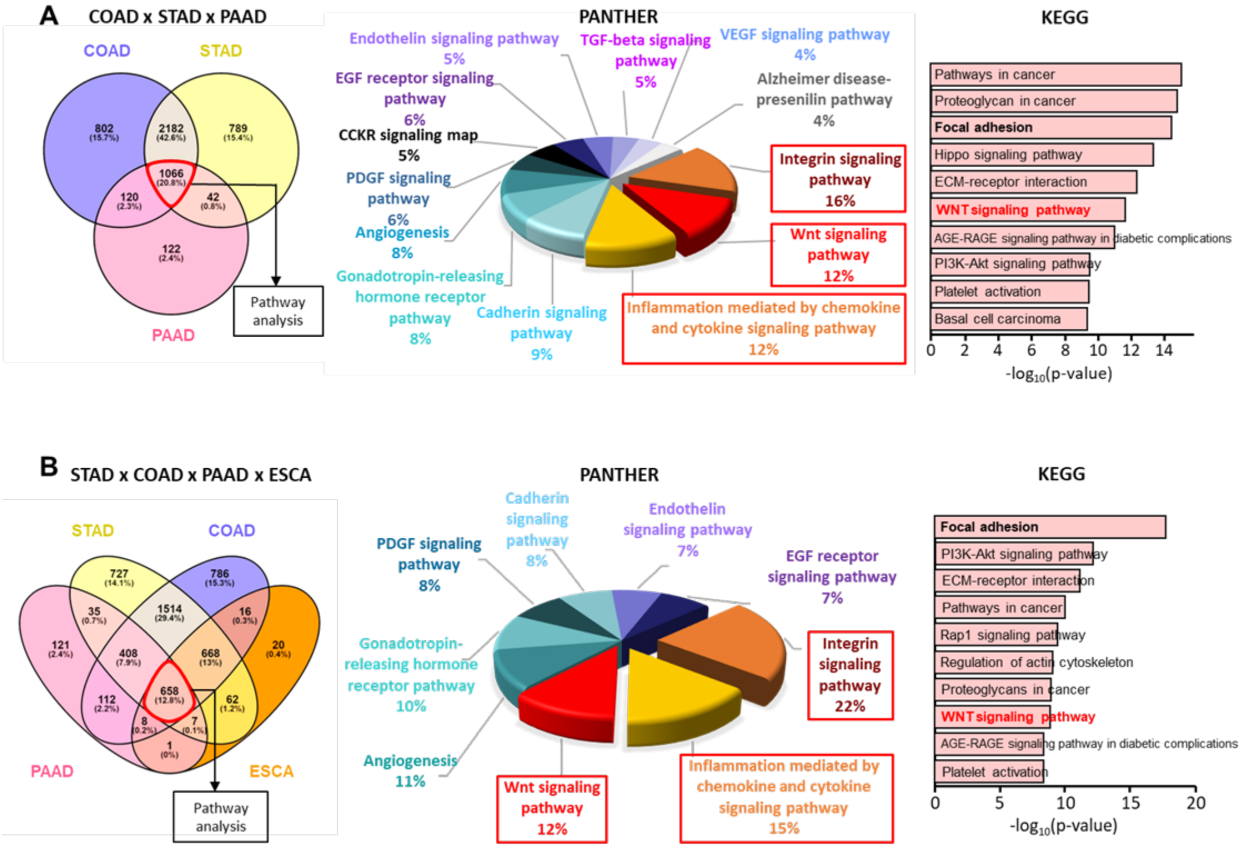

Supplementary Figure S4: The pathway analyses with different cancer combinations. A. and **B.** Venn diagram of genes that positively correlated with LBH in multiple cancer types as indicated (left). The identified common genes from all cancer type combinations were used for PANTHER (middle) of KEGG (right) pathway analysis. Pathways that have at least 10 predicted genes are listed. Note that the top 3 pathways are the integrin, WNT and inflammation signaling throughout different cancer-type combinations. **A.** Analysis of *LBH* positively correlated genres in three cancer types: COAD, STAD, and PAAD, or **B.** four cancer combinations: PAAD, STAD, COAD, and ESCA. COAD; colon adenocarcinoma, STAD; stomach adenocarcinoma, PAAD; pancreatic adenocarcinoma, ESCA; esophageal carcinoma.

Supplementary Figure S5

A

LUAD

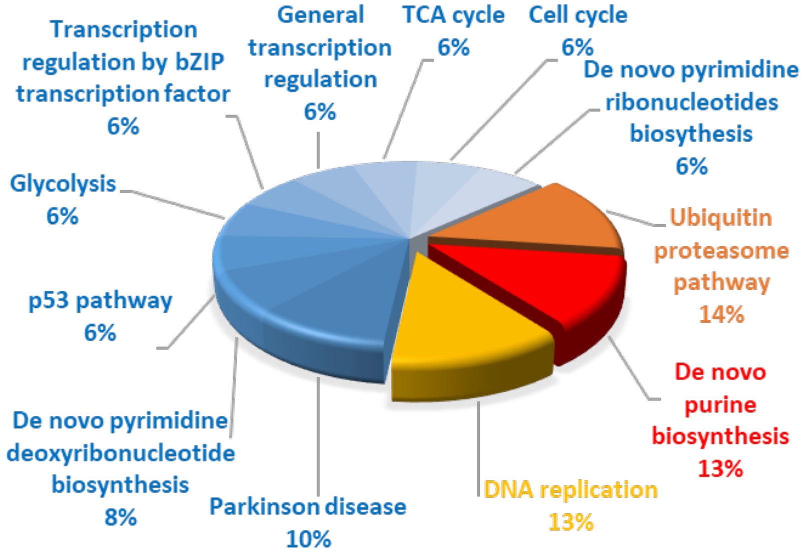

B

SKCM

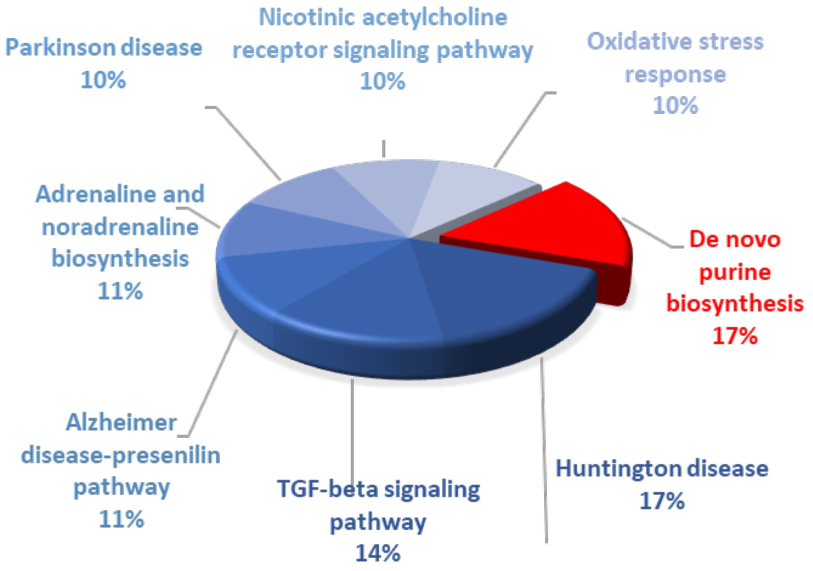

Supplementary Figure S5: LBH correlated genes in LUAD and SKCM. A. Pathway analysis using PANTHER in LUAD. **B.** Pathway analysis using PANTHER in SKCM. (A, B) Pathways that have at least 10 numbers of predicted gene hits are listed. LUAD; lung adenocarcinoma, SKCM; skin cutaneous melanoma.

Supplementary Figure S6

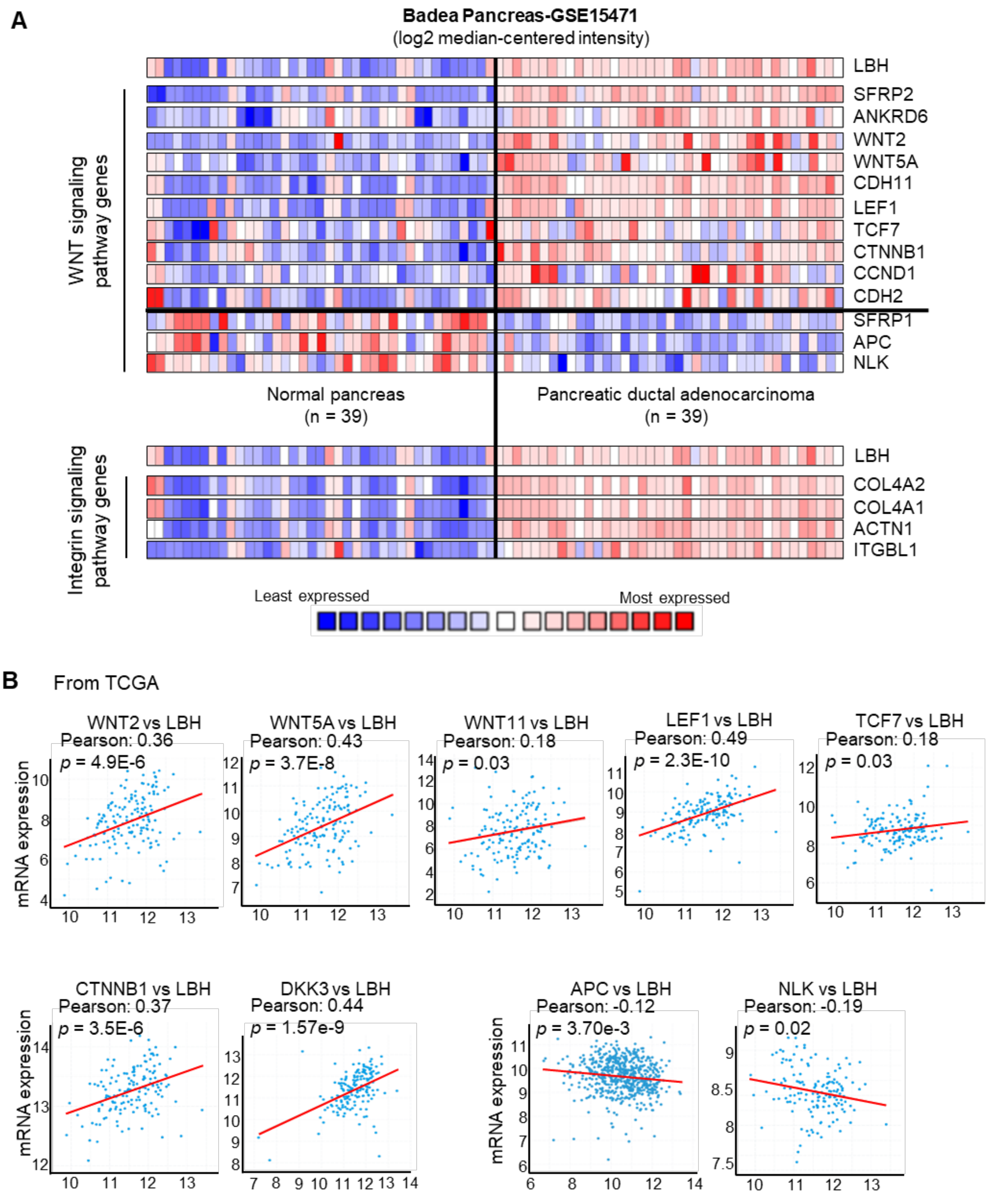

Supplementary Figure S6: *LBH* correlated genes in pancreas cancer. A. Heatmaps of *LBH* and correlated genes in the Wnt and integrin pathways in a representative pancreas cancer dataset (Badea_Pancreas-GSE15471) from Oncomine. Blue signifies low, and red equals high mRNA expression. **B.** Plots represent correlation between LBH and Wnt pathway genes in pancreas cancer. The cBioportal database plotted data derived from TCGA. Pearson correlation coefficients and P-values as indicated.

Supplementary Figure S7

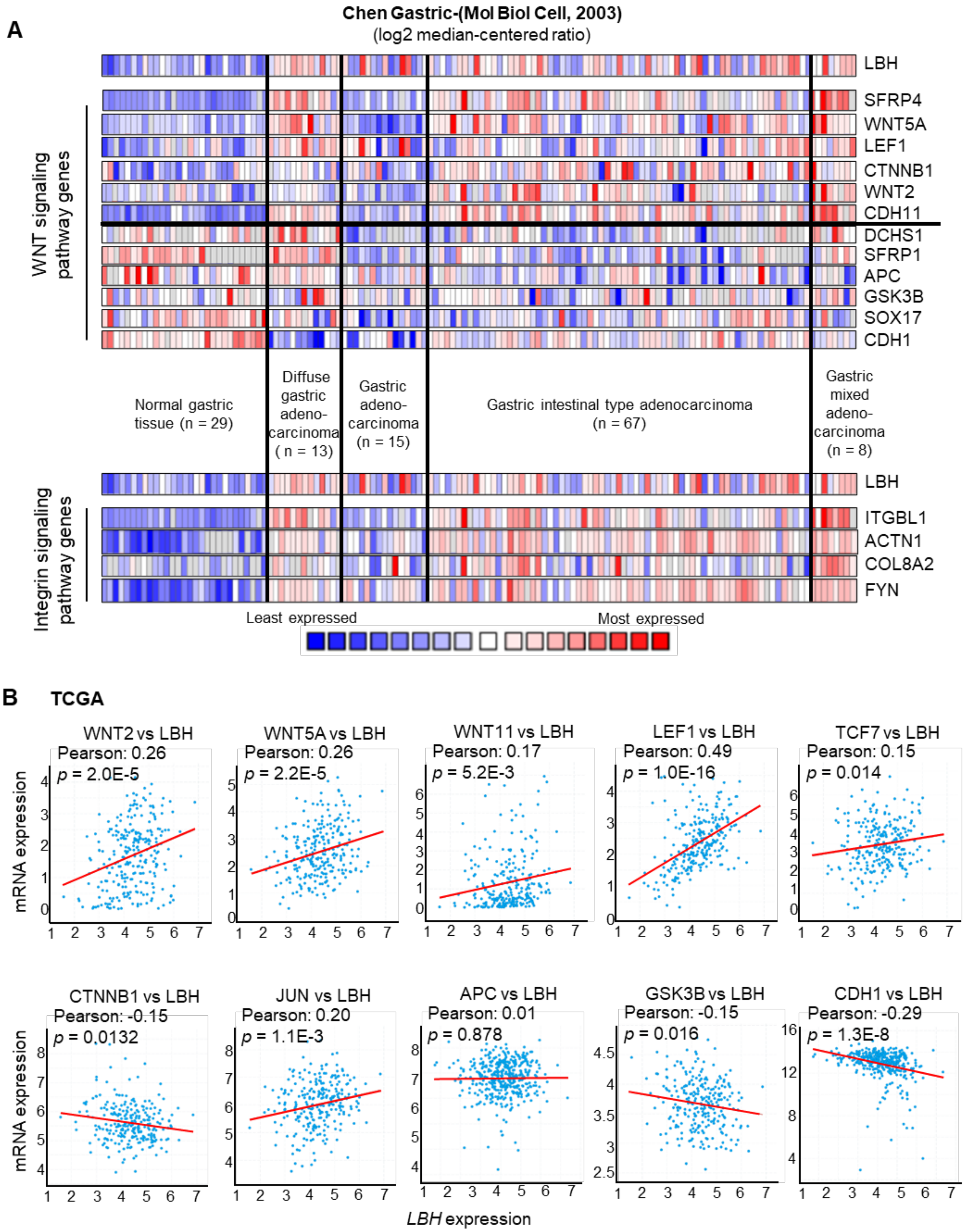

Supplementary Figure S7: *LBH* correlated genes in gastric cancer. A. Heatmaps of *LBH* and correlated genes in the Wnt and integrin pathways in a representative gastric cancer dataset (Chen_Gastric Cancer) from Oncomine. Blue signifies low, and red equals high mRNA expression. **B.** Plots represent correlation between LBH and Wnt pathway genes in gastric cancer. The cBioportal database plotted data derived from TCGA. Pearson correlation coefficients and P-values as indicated.

Supplementary Figure S8

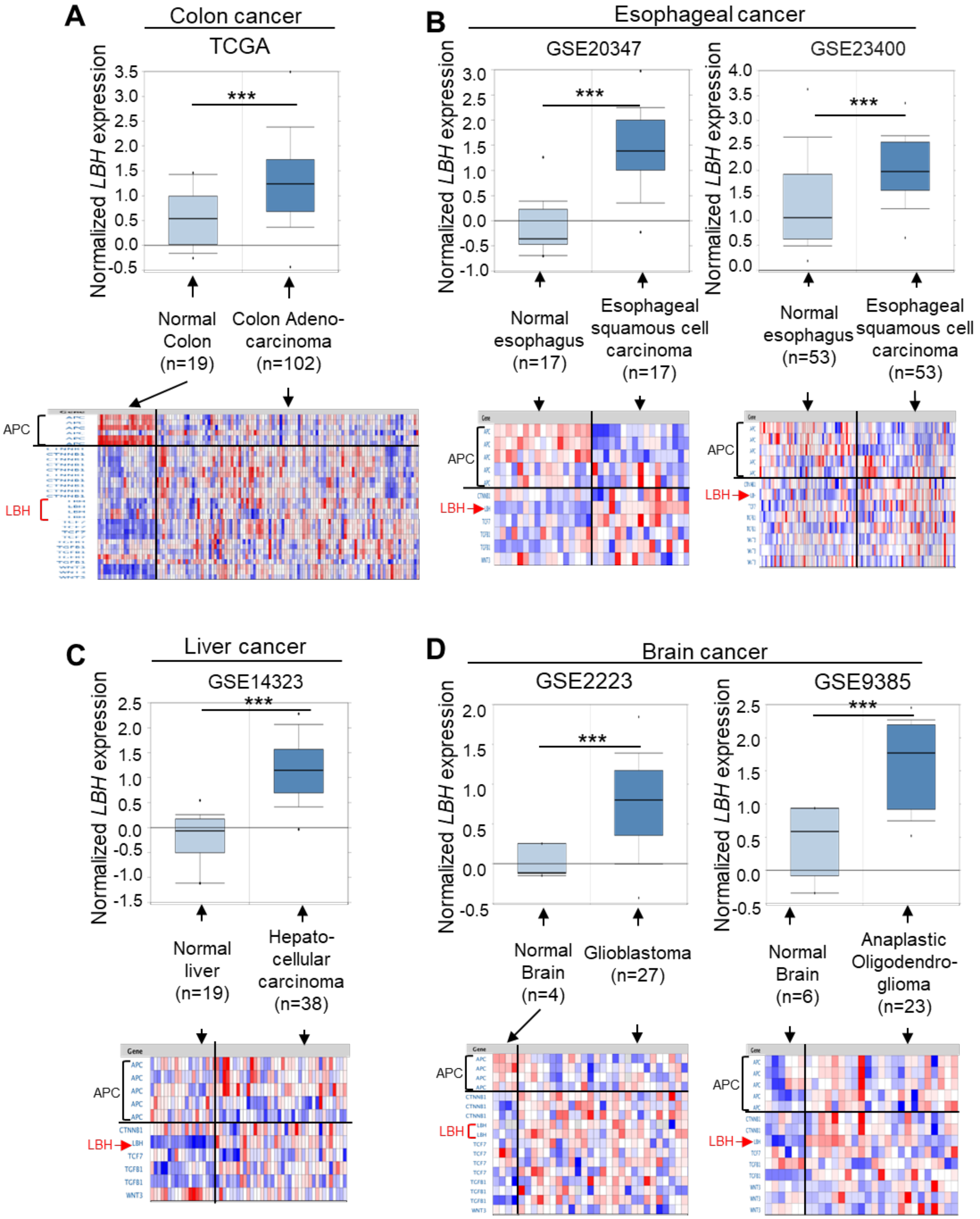

Supplementary Figure S8: Co-expression of *LBH* and WNT pathway genes in different cancer types. A-D, Box plots data showing the fold changes of *LBH* mRNA in tumor (dark blue) versus normal tissues (light blue). Heatmaps of *LBH* and correlated Wnt signaling gene expression (most expressed: red; least expressed: blue) for each patient in the indicted cancer datasets. Patients with *LBH* overexpression showed a high co-expression of oncogenic genes (e.g., *CTNNB1, TCF7, WNT3*), and low co-expression of tumor suppressor genes (e.g., *APC*) in the WNT signaling pathway. Representative human datasets in (**A**) Colon cancer, (**B)** Esophageal cancer, (**C**) Liver cancer, and (**D**) Brain cancer.

SUPPLEMENTARY TABLES

Supplementary Tables S1

Solid tumor cancer types with over-expressed *LBH* compared to normal tissue from the Oncomine database

Cancer	Cancer subtype	*p* -value	Fold change	Rank (%)	Sample	Reference
Brain	Glioblastoma	6.91E-05	1.697	8	54	[1]
	Anaplastic Oligoastrocytoma	0.001	2.188	5	33	[2]
Colorectal	Rectal Adenocarcinoma	5.01E-17	2.315	8	130	[3]
	Colon Carcinoma Epithelia	6.00E-06	2.065	9	40	[4]
Esophagus	Esophageal Adenocarcinoma	6.19E-10	3.919	1	48	[5]
	Esophageal Squamous Cell Carcinoma	5.54E-08	2.907	2	34	[6]
Gastric	Gastric Intestinal Type Adenocarcinoma	1.21E-08	1.577	8	132	[7]
	Diffuse Gastric Adenocarcinoma	4.25E-07	1.816	1	132	[7]
	Gastric Mixed Adenocarcinoma	4.71E-04	1.9	6	132	[7]
Head-Neck	Head and Neck Squamous Cell Carcinoma	3.38E-14	2.013	1	54	[8]
	Oral Cavity Squamous Cell Carcinoma	2.64E-12	1.957	3	79	[9]
	Oral Cavity Squamous Cell Carcinoma Epithelia	9.55E-05	1.92	2	20	[10]
	Oropharyngeal Carcinoma	0.002	1.926	10	84	[11]
Liver	Hepatocellular Carcinoma	1.06E-12	2.532	1	115	[14]
	Cirrhosis	2.54E-12	2.505	5	115	[14]
Pancreas	Pancreatic Ductal Adenocarcinoma	2.96E-11	2.855	3	78	[15]
	Pancreatic Carcinoma	4.66E-04	3.03	5	17	[16]
	Pancreatic Adenocarcinoma	0.001	2.503	5	36	[17]
Prostate	Prostate Carcinoma	0.009	2.001	4	30	[18]
Sarcoma	Myxoid/Round Cell Liposarcoma	5.91E-08	2.864	7	158	[19]
	Myxofibrosarcoma	1.42E-07	2.628	8	158	[19]
	Dedifferentiated Liposarcoma	4.20E-07	2.383	7	158	[19]
	Leiomyosarcoma	1.04E-06	2.623	9	158	[19]
Testis	Testicular Seminoma	1.34E-04	5.602	1	30	[20]
	Testicular Yolk Sac Tumor	0.002	1.999	2	30	[20]
Seminoma	Mixed Germ Cell Tumor, NOS	1.49E-10	3.47	4	107	[21]
	Seminoma, NOS	2.03E-10	5.744	1	107	[21]
	Teratoma, NOS	2.56E-09	4.198	2	107	[21]
	Testicular Seminoma	2.81E-09	2.505	2	74	[22]
	Embryonal Carcinoma, NOS	7.03E-09	4.731	3	107	[21]
	Yolk Sac Tumor, NOS	9.57E-05	2.871	9	107	[21]

Supplementary Tables S2

Solid tumor cancer types with under-expressed *LBH* compared to normal tissue from the Oncomine database

Cancer	Cancer subtype	*p*-value	Fold change	Rank (%)	Sample	Reference
Breast	Invasive Breast Carcinoma	9.31E-05	-2.492	4	158	[24]
	Ductal Breast Carcinoma	2.08E-04	-2.465	10	47	[25]
	Ductal Breast Carcinoma in Situ Epithelia	6.53E-04	-2.285	5	66	[26]
	Invasive Ductal Breast Carcinoma Epithelia	0.001	-1.983	5	66	[26]
	Mucinous Breast Carcinoma	0.004	-2.237	9	593	TCGA
Colorectal	Colon Adenoma Epithelia	2.98E-04	-2.004	9	40	[4]
Lung	Lung Adenocarcinoma	6.39E-17	-2.191	5	116	[29]
	Lung Adenocarcinoma	7.59E-11	-1.714	8	107	[30]
	Large Cell Lung Carcinoma	5.68E-10	-4.381	5	156	[31]
	Lung Adenocarcinoma	1.58E-09	-2.547	3	66	[32]
Melanoma	Cutaneous Melanoma	1.06E-06	-2.059	5	70	[35]

Supplementary Tables S3

Hematopoietic malignancies with differential *LBH* over- or under-expression compared to normal tissue from the Oncomine database.

Cancer	*LBH* expression	Cancer subtype	*p*-value	Fold change	Rank (%)	Sample	Reference
Leukemia	Up	B-Cell Acute Lymphoblastic Leukemia	3.43E-26	37.682	1	127	[12]
		Chronic Lymphocytic Leukemia	3.73E-05	2.789	5	102	[13]
	Down	Acute Myeloid Leukemia	7.47E-26	-2.229	2	2,096	[27]
		Chronic Myelogenous Leukemia	2.65E-24	-2.471	1	2,096	[27]
		Chronic Adult T-Cell Leukemia/Lymphoma	0.008	-2.274	10	47	[28]
Lymphoma	Up	Follicular Lymphoma	1.87E-04	3.446	2	102	[13]
	Down	Anaplastic Large Cell Lymphoma, ALK-Negative	7.56E-13	-3.851	1	64	[33]
		Anaplastic Large Cell Lymphoma	2.81E-05	-2.348	10	60	[34]
		Anaplastic Large Cell Lymphoma, ALK-Positive	3.52E-05	-3.874	3	64	[33]
		Primary Cutaneous Anaplastic Large Cell Lymphoma	1.63E-04	-5.143	5	64	[33]
		Classical Hodgkin's Lymphoma	8.53E-04	-3.085	5	64	[33]
		Chronic Adult T-Cell Leukemia/Lymphoma	0.008	-2.274	10	47	[28]

Supplementary Table S4

The significant prognostic values of *LBH* CpG methylation in cancers

Cancer type	CpG Name	Hazard ratio	CI	Log Rank test P value	UCSC Ref Gene Group	Relation to UCSC CpG Island
STAD	cg20662616	0.548	0.395; 0.76	2.70E-04	Body	Island
	cg04092762	0.519	0.332; 0.81	1.90E-03	TSS1500	Island
	cg08033284	0.603	0.436; 0.834	2.70E-03	TSS200	Island
	cg03801871	0.567	0.368; 0.872	6.00E-03	TSS1500	N_Shore
	cg02233216	0.629	0.443; 0.894	7.90E-03	TSS200	Island
	cg04253876	0.578	0.373; 0.897	9.50E-03	TSS1501	Island
	cg17250262	1.541	1.086; 2.187	0.013	Body	S_Shore
	cg13460277	0.632	0.432; 0.924	0.014	TSS200	Island
	cg04254242	1.529	1.079; 2.167	0.015	3'UTR	Open_Sea
	cg25072436	0.675	0.487; 0.935	0.017	1st Exon; 5'UTR	Island
	cg04683740	0.631	0.421; 0.947	0.020	Body	Open_Sea
	cg05247661	0.66	0.459; 0.949	0.021	Body	Open_Sea
	cg02547025	0.687	0.487; 0.97	0.030	TSS200	Island
	cg09682913	0.7	0.506; 0.968	0.030	Body	Island
	cg26176018	1.526	1.022; 2.278	0.032	Body	Open_Sea
	cg26571942	0.668	0.449, 0.994	0.039	Body	Island
	cg07543711	1.472	1.031; 2.102	0.039	3'UTR	Open_Sea
	cg01626477	0.689	0.473; 1.004	0.046	TSS200	Island
COAD	cg04254242	1.819	1.038; 3.189	0.028	3'UTR	Open_Sea
	cg26571942	0.61	0.379; 0.983	0.041	Body	Island
PAAD	cg04253876	2.172	1.361; 3.465	5.80E-04	TSS1501	Island
	cg25072436	0.635	0.422; 0.957	0.034	1st Exon; 5'UTR	Island
	cg17250262	0.611	0.374; 1	0.04	Body	S_Shore
ESCA	cg04683740	0.533	0.337; 0.842	0.009	Body	Open_Sea
	cg04254242	0.56	0.353; 0.888	0.012	3'UTR	Open_Sea
	cg02233216	0.603	0.363; 1.002	4.40E-02	TSS200	Island
	cg13460277	0.602	0.36; 1.006	0.044	TSS200	Island
	cg07543711	0.61	0.379; 0.981	0.047	3'UTR	Open_Sea

Supplementary Table S5

PANTHER pathway analysis of positively *LBH* co-expressed genes in different cancer types

Gastrointestinal Tract Cancers

COAD

Rank	Category name	genes hit #	genes hit %
1	WNT signaling pathway	111	11.65
2	Inflammation mediated by chemokine and cytokine signaling pathway	108	11.44
3	Gonadotropin-releasing hormone receptor pathway	86	9.11
4	Integrin signaling pathway	87	9.11
5	Cadherin signaling pathway	75	7.84
6	Angiogenesis	66	6.99
7	CCKR signaling map	57	5.93
8	Heterotrimeric G-protein signaling pathway-Gi alpha and Gs alpha mediated pathway	56	5.93
9	PDGF signaling pathway	52	5.51
10	Alzheimer disease-presenilin pathway	50	5.3
11	Heterotrimeric G-protein signaling pathway-Gq alpha and Go alpha mediated pathway	49	5.08
12	EGF receptor signaling pathway	46	4.87
13	T cell activation	39	4.03
14	Apoptosis signaling pathway	35	3.6
15	TGF-beta signaling pathway	35	3.6

STAD

Rank	Category name	genes hit #	genes hit %
1	WNT signaling pathway	109	12.11
2	Inflammation mediated by chemokine and cytokine signaling pathway	91	10.13
3	Gonadotropin-releasing hormone receptor pathway	85	9.47
4	Integrin signaling pathway	81	9.03
5	Cadherin signaling pathway	75	8.37
6	Angiogenesis	63	7.05
7	CCKR signaling map	55	6.17
8	Heterotrimeric G-protein signaling pathway-Gi alpha and Gs alpha mediated pathway	55	6.17
9	PDGF signaling pathway	48	5.29
10	Heterotrimeric G-protein signaling pathway-Gq alpha and Go alpha mediated pathway	45	5.07
11	EGF receptor signaling pathway	43	4.85
12	Alzheimer disease-presenilin pathway	40	4.41
13	TGF-beta signaling pathway	38	4.19
14	Endothelin signaling pathway	35	3.96
15	T cell activation	33	3.74

LIHC

Rank	Category name	genes hit #	genes hit %
1	Inflammation mediated by chemokine and cytokine signaling pathway	124	12.3
2	WNT signaling pathway	119	11.9
3	Integrin signaling pathway	97	9.68
4	Angiogenesis	84	8.27
5	Cadherin signaling pathway	73	7.26
6	Gonadotropin-releasing hormone receptor pathway	69	6.85
7	CCKR signaling map	55	5.44
8	EGF receptor signaling pathway	55	5.44
9	PDGF signaling pathway	53	5.24
10	Alzheimer disease-presenilin pathway	51	5.04
11	T cell activation	49	4.84
12	Heterotrimeric G-protein signaling pathway-Gq alpha and Go alpha mediated pathway	47	4.64
13	Apoptosis signaling pathway	44	4.44
14	Heterotrimeric G-protein signaling pathway-Gi alpha and Gs alpha mediated pathway	44	4.44
15	Huntington disease	43	4.23

Supplementary Table S5 - continued

READ

Rank	Category name	genes hit #	genes hit %
1	Integrin signaling pathway	56	13.23
2	WNT signaling pathway	46	11.02
3	Inflammation mediated by chemokine and cytokine signaling pathway	45	10.62
4	Gonadotropin-releasing hormone receptor pathway	37	8.82
5	Angiogenesis	36	8.62
6	Cadherin signaling pathway	28	6.61
7	Alzheimer disease-presenilin pathway	24	5.61
8	CCKR signaling map	23	5.41
9	PDGF signaling pathway	21	5.01
10	Heterotrimeric G-protein signaling pathway-Gq alpha and Go alpha mediated pathway	20	4.81
11	Heterotrimeric G-protein signaling pathway-Gi alpha and Gs alpha mediated pathway	19	4.61
12	EGF receptor signaling pathway	18	4.21
13	T cell activation	16	3.81
14	FGF signaling pathway	16	3.81
15	Endothelin signaling pathway	16	3.81

PAAD

Rank	Category name	genes hit #	genes hit %
1	Integrin signaling pathway	56	14.36
2	WNT signaling pathway	42	10.73
3	Inflammation mediated by chemokine and cytokine signaling pathway	40	10.18
4	Gonadotropin-releasing hormone receptor pathway	31	8
5	Cadherin signaling pathway	31	8
6	Angiogenesis	30	7.64
7	PDGF signaling pathway	24	6.18
8	EGF receptor signaling pathway	24	6.18
9	CCKR signaling map	19	4.91
10	Endothelin signaling pathway	19	4.91
11	Alzheimer disease-presenilin pathway	17	4.36
12	TGF-beta signaling pathway	17	4.36
13	VEGF signaling pathway	15	3.82
14	T cell activation	13	3.27
15	Apoptosis signaling pathway	12	3.09

ESCA

Rank	Category name	genes hit #	genes hit %
1	Integrin signaling pathway	48	13.16
2	Inflammation mediated by chemokine and cytokine signaling pathway	40	11.13
3	WNT signaling pathway	33	9.11
4	Gonadotropin-releasing hormone receptor pathway	31	8.5
5	Angiogenesis	26	7.09
6	PDGF signaling pathway	22	6.07
7	Heterotrimeric G-protein signaling pathway-Gi alpha and Gs alpha mediated pathway	22	6.07
8	Cadherin signaling pathway	21	5.87
9	Endothelin signaling pathway	20	5.47
10	Heterotrimeric G-protein signaling pathway-Gq alpha and Go alpha mediated pathway	19	5.26
11	EGF receptor signaling pathway	18	5.06
12	CCKR signaling map	17	4.66
13	Alzheimer disease-presenilin pathway	16	4.45
14	T cell activation	16	4.45
15	FGF signaling pathway	13	3.64

Supplementary Table S5 - continued

Urogenital Tract Cancers

KIRC

Rank	Category name	genes hit #	genes hit %
1	WNT signaling pathway	79	10.61
2	Integrin signaling pathway	77	10.41
3	Inflammation mediated by chemokine and cytokine signaling pathway	77	10.41
4	Gonadotropin-releasing hormone receptor pathway	65	8.78
5	Angiogenesis	65	8.78
6	CCKR signaling map	45	6.12
7	PDGF signaling pathway	46	6.12
8	Cadherin signaling pathway	44	5.92
9	Heterotrimeric G-protein signaling pathway-Gi alpha and Gs alpha mediated pathway	41	5.51
10	Alzheimer disease-presenilin pathway	38	5.1
11	EGF receptor signaling pathway	38	5.1
12	Heterotrimeric G-protein signaling pathway-Gq alpha and Go alpha mediated pathway	35	4.69
13	TGF-beta signaling pathway	31	4.29
14	Apoptosis signaling pathway	30	4.08
15	Endothelin signaling pathway	30	4.08

BLCA

Rank	Category name	genes hit #	genes hit %
1	Inflammation mediated by chemokine and cytokine signaling pathway	100	14.07
2	Integrin signaling pathway	79	10.99
3	WNT signaling pathway	60	8.35
4	Angiogenesis	56	7.91
5	Gonadotropin-releasing hormone receptor pathway	55	7.69
6	Heterotrimeric G-protein signaling pathway-Gi alpha and Gs alpha mediated pathway	48	6.81
7	Alzheimer disease-presenilin pathway	46	6.37
8	CCKR signaling map	41	5.71
9	Heterotrimeric G-protein signaling pathway-Gq alpha and Go alpha mediated pathway	39	5.49
10	PDGF signaling pathway	36	5.05
11	T cell activation	34	4.84
12	Cadherin signaling pathway	35	4.84
13	Endothelin signaling pathway	31	4.4
14	EGF receptor signaling pathway	27	3.74
15	Apoptosis signaling pathway	26	3.74

PRAD

Rank	Category name	genes hit #	genes hit %
1	Integrin signaling pathway	24	21.05
2	Angiogenesis	18	15.79
3	Gonadotropin-releasing hormone receptor pathway	13	11.46
4	WNT signaling pathway	13	11.46
5	PDGF signaling pathway	12	10.53
6	Inflammation mediated by chemokine and cytokine signaling pathway	12	10.53
7	EGF receptor signaling pathway	12	10.53
8	T cell activation	10	8.67

Brain Cancer

GBM

Rank	Category name	genes hit #	genes hit %
1	Integrin signaling pathway	17	30.56
2	WNT signaling pathway	14	22.22
3	Alzheimer disease-presenilin pathway	13	19.44
4	CCKR signaling map	12	16.67
5	PDGF signaling pathway	10	11.11

Supplementary Table S6

LBH plus correlated genes in WNT and integrin pathways increased predictive power for cancer survival in multivariate Cox regression analysis using TCGA datasets

COAD

Gene/cluster	*p* value	Hazard ratio	95% CI
LBH	0.015	1.8	1.00 - 2.58
Correlated genes in WNT pathway	1.73E-04	2.54	1.54 - 4.17
Correlated genes in integrin pathway	1.42E-06	3.77	2.17 - 6.50
LBH + correlated genes in WNT pathway	1.85E-04	2.56	1.56 - 4.19
LBH + correlated genes in integrin pathway	1.51E-06	3.79	2.20 - 6.51
LBH + correlated genes in WNT& Integrin pathways	2.30E-09	7.72	5.10 - 12.21

PAAD

Gene/cluster	*p* value	Hazard ratio	95% CI
LBH	0.013	1.89	0.95 - 3.76
Correlated genes in WNT pathway	1.05E-05	2.69	1.72 - 4.19
Correlated genes in integrin pathway	8.21E-07	3.15	1.99 - 5.00
LBH + correlated genes in WNT pathway	1.07E-05	2.7	1.73 - 4.20
LBH + correlated genes in integrin pathway	8.29E-07	3.18	2.01 - 5.03
LBH + correlated genes in WNT& Integrin pathways	6.61E-14	7.41	4.39 - 12.50

STAD

Gene/cluster	*p* value	Hazard ratio	95% CI
LBH	3.00E-03	1.68	1.18 - 2.4
Correlated genes in WNT pathway	3.44E-06	2.37	1.63 - 3.43
Correlated genes in integrin pathway	1.78E-04	2.55	1.55 - 4.18
LBH + correlated genes in WNT pathway	3.65E-06	2.39	1.65 - 3.45
LBH + correlated genes in integrin pathway	1.85E-04	2.56	1.56 - 4.19
LBH + correlated genes in WNT& Integrin pathways	1.62E-08	5.95	3.20 - 11.05

Statistical analysis: log-rank test

Supplementary Table S7: Primers

Primers used for Methylation-specific qPCR

Primer set	Forward	Reverse
LBH-M1 (Methylation product 1)	GTTTTTTGTTTTTGTTGTTATGAGC	CTAATCTACAACCCTAAATTCCGTA
LBH-U1 (Unmethylation product 1)	TTTTTGTTTTTGTTGTTATGAGTGA	TAATCTACAACCCTAAATTCCATA
LBH-M2	TTTTTCGTAAGTTAACGCGTTTC	TAAACCTCTCTCTAAAATCCTCGC
LBH-U2	AGTTTTTTGTAAGTTAATGTGTTTTGT	TAAACCTCTCTCTAAAATCCTCACC
LBH-M3	GAGGTTTAGTATGGGGAATAAACGT	GCTCGAAACATAACAACTCGAC
LBH-U3	GAGGTTTAGTATGGGGAATAAATGT	AAACCACTCAAAACATAACAACTCA

Primers used for qPCR

Primer set	Forward	Reverse
LBH	TCACTGCCCCGACTATCTG	GGTTCCACCACTATGGAGG
GAPDH	GGTTCCACCACTATGGAGG	GACAAGCTTCCCGTTCTCAG

SUPPLEMENTARY REFERENCES

1 Bredel, M., Functional Network Analysis Reveals Extended Gliomagenesis Pathway Maps and Three Novel MYC-Interacting Genes in Human Gliomas. 2005. 65(19): p. 8679-8689.

2 French, P.J., et al., Gene Expression Profiles Associated with Treatment Response in Oligodendrogliomas. Cancer Research, 2005. 65(24): p. 11335-11344.

3 Gaedcke, J., et al., Mutated KRAS results in overexpression of DUSP4, a MAP-kinase phosphatase, and SMYD3, a histone methyltransferase, in rectal carcinomas. Genes, Chromosomes and Cancer, 2010. 49(11): p. 1024-1034.

4 Skrzypczak, M., et al., Modeling Oncogenic Signaling in Colon Tumors by Multidirectional Analyses of Microarray Data Directed for Maximization of Analytical Reliability. PLoS ONE, 2010. 5(10): p. e13091.

5 Hao, Y., et al., Gene expression profiling reveals stromal genes expressed in common between Barrett's esophagus and adenocarcinoma. Gastroenterology, 2006. 131(3): p. 925-33.

6 Hu, N., et al., Genome wide analysis of DNA copy number neutral loss of heterozygosity (CNNLOH) and its relation to gene expression in esophageal squamous cell carcinoma. BMC Genomics, 2010. 11(1): p. 576.

7 Chen, X., et al., Variation in Gene Expression Patterns in Human Gastric Cancers. Molecular Biology of the Cell, 2003. 14(8): p. 3208-3215.

8 Ginos, M.A., et al., Identification of a gene expression signature associated with recurrent disease in squamous cell carcinoma of the head and neck. Cancer Res, 2004. 64(1): p. 55-63.

9 Peng, C.H., et al., A novel molecular signature identified by systems genetics approach predicts prognosis in oral squamous cell carcinoma. PLoS One, 2011. 6(8): p. e23452.

10 Toruner, G.A., et al., Association between gene expression profile and tumor invasion in oral squamous cell carcinoma. Cancer Genet Cytogenet, 2004. 154(1): p. 27-35.

11 Pyeon, D., et al., Fundamental differences in cell cycle deregulation in human papillomavirus-positive and human papillomavirus-negative head/neck and cervical cancers. Cancer Res, 2007. 67(10): p. 4605-19.

12 Andersson, A., et al., Microarray-based classification of a consecutive series of 121 childhood acute leukemias: prediction of leukemic and genetic subtype as well as of minimal residual disease status. Leukemia, 2007. 21(6): p. 1198-203.

13 Rosenwald, A., et al., Relation of gene expression phenotype to immunoglobulin mutation genotype in B cell chronic lymphocytic leukemia. J Exp Med, 2001. 194(11): p. 1639-47.

14 Mas, V.R., et al., Genes involved in viral carcinogenesis and tumor initiation in hepatitis C virus-induced hepatocellular carcinoma. Mol Med, 2009. 15(3-4): p. 85-94.

15 Wurmbach, E., et al., Genome-wide molecular profiles of HCV-induced dysplasia and hepatocellular carcinoma. Hepatology, 2007. 45(4): p. 938-47.

16 Badea, L., et al., Combined gene expression analysis of whole-tissue and microdissected pancreatic ductal adenocarcinoma identifies genes specifically overexpressed in tumor epithelia. Hepatogastroenterology, 2008. 55(88): p. 2016-27.

17 Segara, D., et al., Expression of HOXB2, a retinoic acid signaling target in pancreatic cancer and pancreatic intraepithelial neoplasia. Clin Cancer Res, 2005. 11(9): p. 3587-96.

18 Iacobuzio-Donahue, C.A., et al., Exploration of global gene expression patterns in pancreatic adenocarcinoma using cDNA microarrays. Am J Pathol, 2003. 162(4): p. 1151-62.

19 Luo, J.H., et al., Gene expression analysis of prostate cancers. Mol Carcinog, 2002. 33(1): p. 25-35.
20 Barretina, J., et al., Subtype-specific genomic alterations define new targets for soft-tissue sarcoma therapy. Nat Genet, 2010. 42(8): p. 715-21.
21 Skotheim, R.I., et al., Differentiation of human embryonal carcinomas in vitro and in vivo reveals expression profiles relevant to normal development. Cancer Res, 2005. 65(13): p. 5588-98.
22 Korkola, J.E., et al., Down-regulation of stem cell genes, including those in a 200-kb gene cluster at 12p13.31, is associated with in vivo differentiation of human male germ cell tumors. Cancer Res, 2006. 66(2): p. 820-7.
23 Sperger, J.M., et al., Gene expression patterns in human embryonic stem cells and human pluripotent germ cell tumors. Proc Natl Acad Sci U S A, 2003. 100(23): p. 13350-5.
24 Glück, S., et al., TP53 genomics predict higher clinical and pathologic tumor response in operable early-stage breast cancer treated with docetaxel-capecitabine ± trastuzumab. Breast Cancer Res Treat, 2012. 132(3): p. 781-91.
25 Ma, X.J., et al., Gene expression profiling of the tumor microenvironment during breast cancer progression. Breast Cancer Res, 2009. 11(1): p. R7.
26 Richardson, A.L., et al., X chromosomal abnormalities in basal-like human breast cancer. Cancer Cell, 2006. 9(2): p. 121-32.
27 Haferlach, T., et al., Clinical utility of microarray-based gene expression profiling in the diagnosis and subclassification of leukemia: report from the International Microarray Innovations in Leukemia Study Group. J Clin Oncol, 2010. 28(15): p. 2529-37.
28 Choi, Y.L., et al., A genomic analysis of adult T-cell leukemia. Oncogene, 2007. 26(8): p. 1245-55.
29 Su, L.J., et al., Selection of DDX5 as a novel internal control for Q-RT-PCR from microarray data using a block bootstrap re-sampling scheme. BMC Genomics, 2007. 8: p. 140.
30 Selamat, S.A., et al., Genome-scale analysis of DNA methylation in lung adenocarcinoma and integration with mRNA expression. Genome Res, 2012. 22(7): p. 1197-211.
31 Hou, J., et al., Gene expression-based classification of non-small cell lung carcinomas and survival prediction. PLoS One, 2010. 5(4): p. e10312.
32 Landi, M.T., et al., Gene expression signature of cigarette smoking and its role in lung adenocarcinoma development and survival. PLoS One, 2008. 3(2): p. e1651.
33 Eckerle, S., et al., Gene expression profiling of isolated tumour cells from anaplastic large cell lymphomas: insights into its cellular origin, pathogenesis and relation to Hodgkin lymphoma. Leukemia, 2009. 23(11): p. 2129-38.
34 Piccaluga, P.P., et al., Gene expression analysis of peripheral T cell lymphoma, unspecified, reveals distinct profiles and new potential therapeutic targets. J Clin Invest, 2007. 117(3): p. 823-34.
35 Talantov, D., et al., Novel genes associated with malignant melanoma but not benign melanocytic lesions. Clin Cancer Res, 2005. 11(20): p. 7234-42.
